# Learning and memory impairments in a neuroendocrine mouse model of anxiety/depression

**DOI:** 10.3389/fnbeh.2014.00136

**Published:** 2014-05-01

**Authors:** Flavie Darcet, Indira Mendez-David, Laurent Tritschler, Alain M. Gardier, Jean-Philippe Guilloux, Denis J. David

**Affiliations:** EA3544, Neuropharmacologie des troubles anxio-dépressifs et neurogenèse, Faculté de Pharmacie, Université Paris-SudChâtenay-Malabry, France

**Keywords:** depression, anxiety/depression model, corticosterone, recognition memory, spatial learning maze, associative memory, cognitive impairments, cognitive flexibility

## Abstract

Cognitive disturbances are often reported as serious incapacitating symptoms by patients suffering from major depressive disorders (MDDs). Such deficits have been observed in various animal models based on environmental stress. Here, we performed a complete characterization of cognitive functions in a neuroendocrine mouse model of depression based on a chronic (4 weeks) corticosterone administration (CORT). Cognitive performances were assessed using behavioral tests measuring episodic (novel object recognition test, NORT), associative (one-trial contextual fear conditioning, CFC), and visuo-spatial (Morris water maze, MWM; Barnes maze, BM) learning/memory. Altered emotional phenotype after chronic corticosterone treatment was confirmed in mice using tests predictive of anxiety or depression-related behaviors. In the NORT, CORT-treated mice showed a decrease in time exploring the novel object during the test session and a lower discrimination index compared to control mice, characteristic of recognition memory impairment. Associative memory was also impaired, as observed with a decrease in freezing duration in CORT-treated mice in the CFC, thus pointing out the cognitive alterations in this model. In the MWM and in the BM, spatial learning performance but also short-term spatial memory were altered in CORT-treated mice. In the MWM, unlike control animals, CORT-treated animals failed to learn a new location during the reversal phase, suggesting a loss of cognitive flexibility. Finally, in the BM, the lack of preference for the target quadrant during the recall probe trial in animals receiving corticosterone regimen demonstrates that long-term retention was also affected in this paradigm. Taken together, our results highlight that CORT-induced anxio-depressive-like phenotype is associated with a cognitive deficit affecting all aspects of memory tested.

## Introduction

The prevalence of depression, a severe psychiatric disease, is constantly high worldwide to the extent that World Health Organization (WHO) estimates that Major Depressive Disorder (MDD) will be the second largest cause of disability in year 2020 (WHO, 2008). Major depression is characterized by a set of emotional and behavioral alterations, including persistent depressed mood and loss of interest or pleasure as core symptoms. Since cognitive symptoms are common among patients with MDD (Fava et al., 2006; Hammar and Ardal, 2009; Murrough et al., 2011; Lee et al., 2012; Millan et al., 2012), investigators have examined the nature of difficulties in cognitive functioning that are associated with depression such as attention (Landro et al., 2001; Ravnkilde et al., 2002; Porter et al., 2003; Lampe et al., 2004), processing speed (Ravnkilde et al., 2002; Hammar et al., 2003; Lampe et al., 2004), executive function (Naismith et al., 2003; Lampe et al., 2004) and learning and memory (Landro et al., 2001; Fossati et al., 2002; Ravnkilde et al., 2002; Porter et al., 2003; Vythilingam et al., 2004). A recent literature review e assessed abnormalities in neural circuits and cognition early in the course of MDD (Trivedi and Greer, 2014). Interestingly, cognitive deficits in memory and decision-making are detected early in the course of MDD and may be associated with structural abnormalities in the hippocampus or cortex (Trivedi and Greer, 2014). New antidepressant drug strategies that also target cognitive symptoms are needed to improve long-term outcomes, particularly functional recovery.

In order for basic research to provide potential advances in the field, it is critical to use animal models that present behavioral, neurochemical and brain morphological phenotype reminiscent of some symptoms of depression including cognitive impairments. A variety of studies have assessed cognitive disorders in anxiety or depression models in rodents (Patki et al., 2013; Richter et al., 2013). However, some of these only focused their work on a single aspect of learning and memory. Cognitive dysfunctions in Chronic Mild Stress-exposed rats (CMS) have been shown in several behavioral paradigms. For example, CMS induces spatial learning and memory impairments in the Morris water maze (MWM) test in mice (Song et al., 2006), recognition memory deficits in both rats (Orsetti et al., 2007) and mice (Elizalde et al., 2008) and suppression of fear extinction (Garcia et al., 2008). Similarly, maternal separation and social defeat models were employed to highlight spatial reference memory deficits (Couto et al., 2012; Patki et al., 2013).

Here, we performed a thorough characterization of cognitive performances in a neuroendocrine mouse model of depression based on a chronic CORT (David et al., 2009; Mendez-David et al., 2014). Given the multiple forms of learning and memory, we selected a range of cognitive behavioral paradigms that allows investigation of different memory systems including short-term episodic memory, associative memory and spatial reference learning and memory. Additional parameters such as cognitive flexibility and long-term memory were also evaluated in spatial memory tests.

## Materials and methods

### Animals

Eight to 10-weeks old male C57BL/6J mice (Janvier Labs, France) were maintained on a 12L:12D schedule and were housed 5 per cage. Food and water were provided *ad libitum*. All behavioral testing occurred during the light phase between 7 am and 7 pm and were conducted in compliance with animal cares guidelines and with protocols approved by the Institutional Animal Care and Use Committee (Council Directive #87-848, October 19, 1987, Ministère de l'Agriculture et de la Forêt, Service Vétérinaire de la santé et de la Protection Animale, permission #92-256B to Denis J. David).

### Drugs

**Figure 1:**
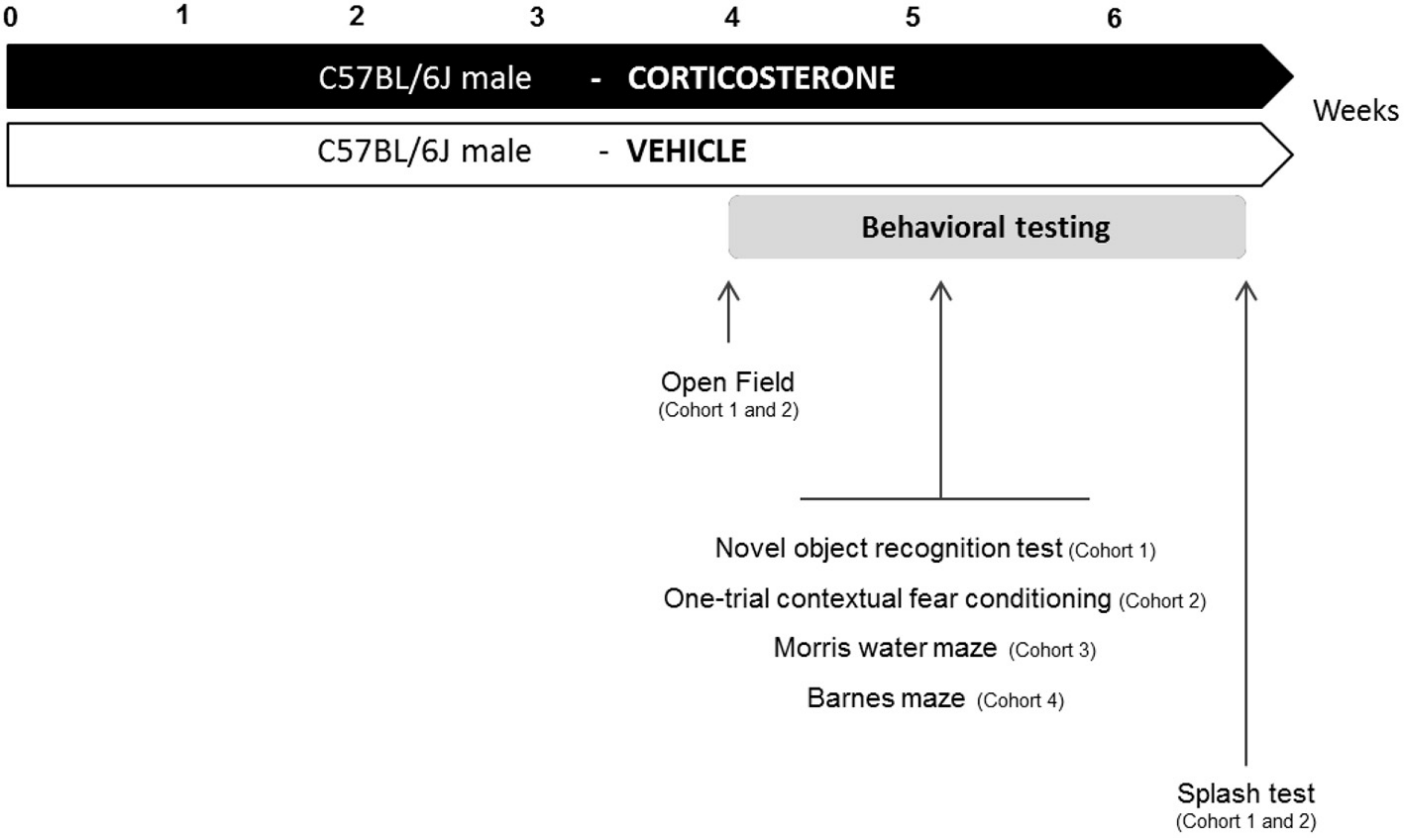
**Experimental design**. In place of normal drinking water, grouped-housed male C57BL/6J mice were presented with vehicle (0.45% hydroxypropyl-β-cyclodextrin) or corticosterone (35 μg/mL). We performed a complete characterization of cognitive functions in four different cohorts of animals. The same animal was successively tested in the Open Field paradigm, the cognitive task (Novel object recognition test: cohort 1; One-trial contextual fear conditioning: cohort 2) and the Splash test. Cohort 3 and 4 were only tested for cognitive tasks (Morris water maze: cohort 3; Barnes maze: cohort 4).

Corticosterone (4-pregnen-11b-diol-3 20-dione 21 hemisuccinate, CORT from Sigma-Aldrich, France) was dissolved in vehicle (0.45% hydroxypropyl-β-cyclodextrin, β-CD from Sigma-Aldrich, France). Corticosterone (35 μg/ml equivalent to 5 mg/kg/day) was delivered for 28 days in drinking water and continued when the behavioral tests were performed (David et al., 2009). Control animals received vehicle (β-CD) in drinking water during the entire experiment (Figure 1).

### Behavioral testing

Four different cohorts of mice were used to assess learning and memory performances in anxio-depressive animals. To prevent any confounding effects between cognitive tasks, each animal was subjected to only one type of learning and memory test. Fur coat state of the animals was scored weekly during the whole treatment period. The anxio-depressive-like phenotype induced by chronic corticosterone among the different cohorts was evaluated using the Open Field and the Splash tests, before and after the cognitive task, respectively. Details of emotional assessment are mentioned in Figure 1.

Cognitive performances were evaluated using behavioral tests measuring episodic (novel object recognition test, NORT), associative (one-trial contextual fear conditioning, CFC) and visuo-spatial (MWM; Barnes maze, BM) learning/memory. Animals were placed in the experimental room 30 min before the start of the behavioral experiments.

### Anxiety and depression behavior paradigms

#### Fur coat state

The score corresponding to the state of the coat resulted from the sum of the score of five different body parts: head, neck, dorsal/ventral coat, tail and fore-/hind paws. For each body area, a score of 0 was given for a well-groomed coat and 1 for an unkempt coat (Surget et al., 2008; David et al., 2009).

#### Open field

Motor activity was quantified in 43 × 43 cm plexiglas open field boxes (MED Associates, Georgia, VT). Two sets of 16 pulse-modulated infrared photobeams were placed on opposite walls 2–5 cm apart to record x-y ambulatory movements. Activity chambers were computer interfaced for data sampling at 100 ms resolution. The computer defined grid lines that divided each open field into center and surround regions, with each of four lines being 11 cm from each wall. Time in the center was recorded for 30 min to evaluate anxiety-related behavior. The locomotor activity was quantified as total ambulatory distance.

#### Splash test

Splash test was performed as previously described (David et al., 2009). This test consisted in squirting a 10% sucrose solution on the mouse's snout. The grooming duration was then recorded over a 5 min period.

### Cognition behavioral paradigms

#### Episodic short-term memory: novel object recognition test

The procedure was adapted from the Sahay study (Sahay et al., 2011). The apparatus consisted in black plastic boxes (28 × 41 × 18 cm) slightly filled with sawdust (≈0.5–1 cm thickness) in a room with a low level of light. Locomotor activity was controlled during the entire experiment (parameter: ambulatory distance) using a videotracking procedure (ANY-maze Software, Bioseb, France). Objects exploration was hand-scored by an experimenter.

**Figure 2:**
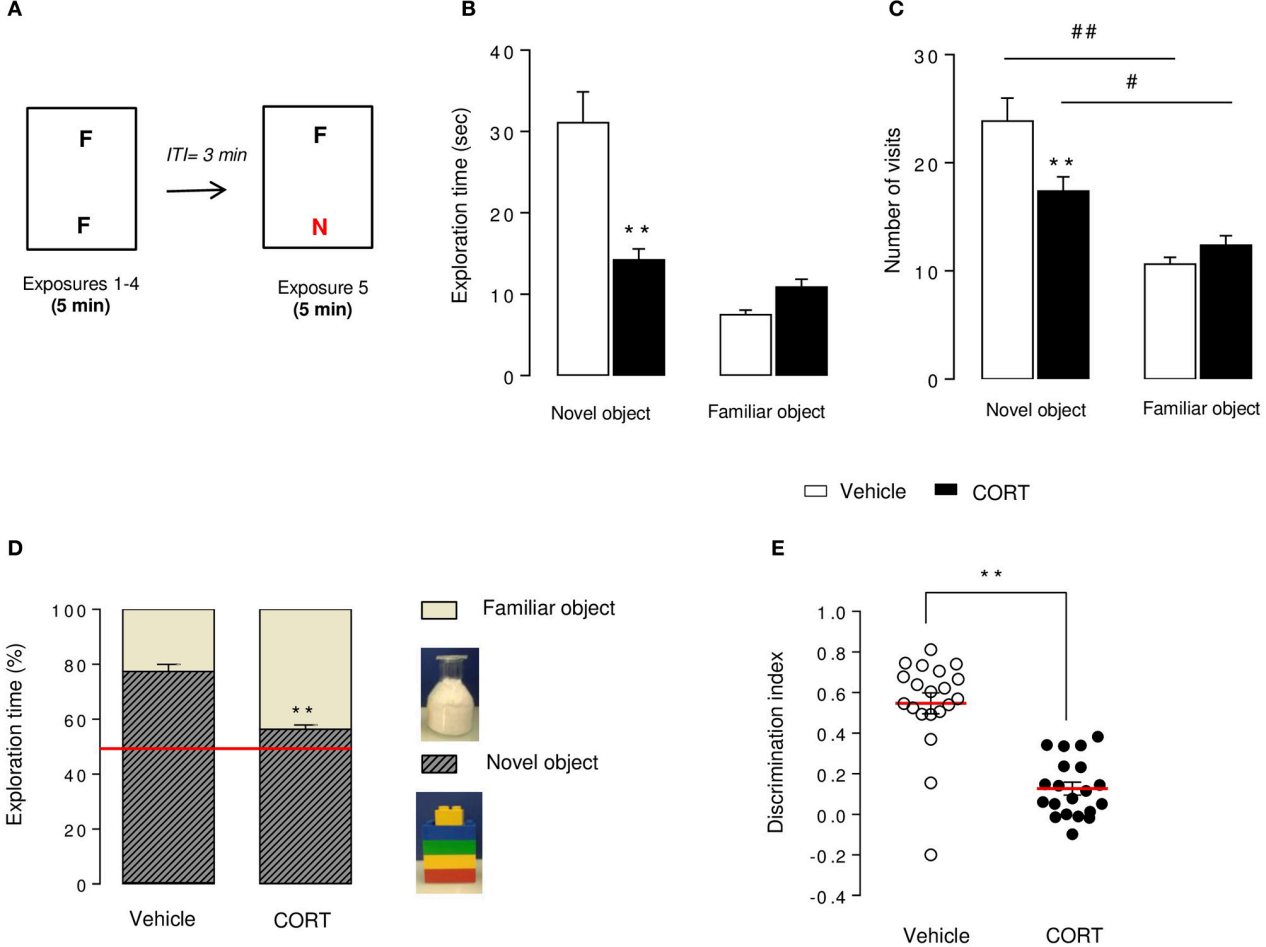
**Short-term episodic memory is altered in CORT-treated mice in the novel object recognition test**. Schematic diagram of the NORT experimental time course **(A)**. Mice were exposed to 2 identical objects during 4 sessions (5 min each) with an intertrial interval (ITI) of 3 min. During the 5thexposure, one familiar object (F) was removed and replaced with a novel object (N) different in shape, color, and texture. Exploration time **(B)** and exploration frequency **(C)** of the novel and the familiar objects were measured during the test session. The exploration time was also expressed in percent of both novel object and familiar object **(D)**. The red line indicates the chance level. Discrimination index **(E)** of each animal was calculated (see Materials and Methods). Values are mean ± s.e.m., *n* = 20 animals per group ^**^p < 0.01 vs. vehicle group. ^#^*p* < 0.05; ^##^*p* < 0.01 vs. novel object of the group.

The NORT was divided into 4 training sessions and one test session. Each exposure lasted 5 min with a 3-min inter-trial interval. Between each trial, mice returned to their home cage, bedding of apparatus was changed and boxes were cleaned with 70% ethanol solution. During training sessions, two identical objects [cylindrical glassware (∅:3 cm, height: 8 cm) filled with white cotton, (Figure 2D)] were present in the box. The mouse was placed in the middle of the box facing the wall and was allowed to freely explore the apparatus and the objects. During the test session, one of the familiar objects was removed from the cage and replaced by a novel object [Lego® rectangular structure (7 × 3 × 9 cm), (Figure 2D)]. The objects had been previously validated to ensure there was no inherent preference for either object (data not shown). The nature (Lego® vs. glass) and the position of the novel object (left vs. right) were chosen randomly.

Object exploration was defined as the orientation of the nose to the object at a distance ≤ 2 cm. Placing the forepaws on the objects was considered as exploratory behavior, but climbing on the objects was not. Objects were cleaned with 70% ethanol between trials to avoid olfactory cues. Results for this test were expressed as: (1) exploration of each object (in seconds) during training and test sessions(2) exploration (in percent) of each object during the test session, calculated as time spent exploring familiar or novel object divided by total time spent exploring both objects and (3) a discrimination index (DI) between objects during the test session, calculated as the difference between the time spent exploring the novel object (N) and the familiar object (F) divided by the total time exploring both objects (DI = (N–F)/(N+F)).

#### Associative memory: one-trial contextual fear conditioning

Fear conditioning was conducted in StartFear Combined system from Harvard apparatus (Bioseb, France) in chambers with internal dimensions of 25 × 25 × 25 cm. The chambers had metal walls on each side, clear plastic front and back walls and ceilings, and stainless steel bars on the floor. A house light mounted directly on the side of the chamber provided illumination. Each chamber was located inside a larger, insulated, plastic cabinet (67 × 53 × 55 cm) that provided protection from outside light and noise. Each cabinet contained a ventilation fan that was operated during the sessions. Mice were held outside the experimental room in their home cages prior to testing and transported to the conditioning apparatus individually in standard mouse cages. Training chambers were cleaned with 70% ethanol solution before and after each trial to avoid any olfactive cues. The experimental design was adapted from previous studies (Drew et al., 2010) and ran over two consecutive days. On Day 1, mice were placed in the conditioning chamber and 3 min later received one shock (2 s, 0.75 mA). Mice were removed from the chamber 15 s after the shock. On Day 2, animals returned to the conditioning chamber for a 4-min period in the exact same conditions of Day 1, but without electrical shock, for a test of context-elicited freezing. Scoring was measured using Freezing software version 2.0.04 (Packwin, Harvard apparatus, Bioseb, France). The StartFear system allows recording and analyzing the signal generated by the animal movement through a high sensitivity weight transducer system. The behavior of mice was also recorded with digital video cameras mounted above the conditioning chamber.

#### Spatial reference learning and memory: morris water maze and barnes maze

***Morris water maze***. The MWM procedure was adapted from (Sahay et al., 2011). The apparatus consists in a circular pool (∅: 122 cm, height: 50 cm) filled with 30 cm-depth water maintained at 22–23°C and made opaque by addition of white milk powder. The maze was divided into four quadrants (North, South, West, and East). Mice learned to locate an unmarked submerged platform in a pool devoid of intramaze cues. Geometrical extra cues were surrounding the maze to generate spatial learning. The escape platform (∅: 10 cm; height: 30 cm) was placed in the target quadrant (North), 1 cm above the surface of water during the pre-training session and 1 cm below the water surface during the other sessions. The MWM task was performed with three successive steps (Figure 4A). The pre-training phase (1 session, 3 trials, Day 0) allowed mice to accustom to the pool and the visible platform placed in clear water. The acquisition phase was divided into 4 training sessions (Day 1–4) and one probe trial (Day 5). During training sessions, the platform was hidden to develop spatial learning. Each mouse received three 60-s trials per day with 60 s inter-trial intervals. The starting points were semi-randomized so that each trial started from a different quadrant. Between each trial, the mouse had to remain on the platform during 60 s. If the mouse did not find the platform within 60 s, the experimenter gently guided the animal to the platform. In this case, 60 s was recorded as the escape latency. A 60-s probe session (Day 5), during which the platform was removed, was performed 24 h after the last trial of the learning period.

The same procedure was applied for the reversal phase in which the hidden platform was located in the opposite quadrant (South) during training sessions (Day 8–11). The platform was removed in the 2nd probe trial (Day 12) to assess spatial retention.

Time spent in target and opposite quadrants, latency to cross the platform zone for the first time and the number of entries in the platform zone were recorded. Mouse movement was measured using a video tracking system and analyzed by ANY-maze Software to record latency, distance and pathways to reach the escape platform through all the sessions.

***Barnes maze***. The BM procedure was modified from a previous work (Sunyer et al., 2007). The apparatus consisted in a clear gray circular platform (∅: 92 cm, height: 100 cm; Bioseb, France) with 20 equally spaced holes (∅: 5 cm) located 2 cm from the border. In this open environment, mice naturally seek a dark enclosed surrounding place, provided by a black goal box (20 × 9 × 9 cm) located beneath one of the holes. During training sessions, the 19 other holes are closed. From the surface of the maze, the open escape hole looks identical to the closed holes so that the mice can locate the target box only with the spatial extra cues surrounding the maze. Similarly to the MWM test, the circular platform was virtually divided in 4 zones (including the target quadrant with the escape hole and the opposite quadrant). To reduce anxiety behavior, mice were habituated to the platform and the target box on the day before the beginning of the experiment.

Each trial began by placing the animal in a black starting cylinder (∅: 8 cm, height: 12.5 cm) at the center of the platform that was removed after 10 s, allowing mice to freely explore the apparatus. Spatial acquisition was organized in 4 training sessions (Day 1–4, Figure 5A). Each training session consisted in four 3-min trials, with 20 min inter-trials interval during which animals returned to their home cage. Mice that failed to find the target box within 3 min were gently guided to its location. For those mice, 180 s were recorded as the escape latency. All animals remained in the target box for 60 s after entering.

All trials were recorded by a camera and analyzed by ANY-maze Software. The following parameters were scored during all training trials: primary latency, latency to escape, primary errors and total errors. Primary latency was defined as the time required for mice to make initial contact with the target hole. Latency to escape was defined as the time it took animals to completely enter in the target box (all 4 paws out of the platform). Primary errors were defined as the number of holes visited before the first contact with the target hole and total errors were defined as the total number of holes visited during the trial that did not lead to the target box. A hole was considered visited when mice tilted their head over it (nose poke) or introduced their paws into the hole.

On Day 5, reference short-term memory was evaluated by a probe trial (90 s) during which the target box was removed and the target hole was closed. Mice were allowed to explore the maze and to visit the target hole and the adjacent holes. Latency to reach the target hole for the first time, number of errors before reaching the target hole, distribution of visits among all holes and time spent in each quadrant were recorded. On Day 12, mice were once again submitted to a probe trial (recall) in the same conditions as Day 5 to evaluate long-term retention. No training occurred between Days 5 and 12.

### Statistics

Results from data analyses were expressed as mean ± s.e.m. Statistical analyses were processed with Statview 5.0® Software (SAS Institute, Cary, NC). For all experiments, comparisons between CORT-treated and control animals were performed by using *t*-tests. One-Way ANOVA with repeated-measures and Two-Way ANOVA were applied to the data when appropriate. Significant main effects and/or interactions were followed by Fisher's PLSD *post-hoc* analysis. One sample *t*-tests were used to compare the percent of time exploring the novel object vs. the chance level (50%) in the NORT and the time spent in the target quadrant vs. the chance level (25%) in the MWM and BM tests. For the latency to cross the platform in reversal phase, we used the Kaplan-Meier survival analysis due to the lack of normal distribution of the data. Mantel-Cox log-rank test was used to evaluate differences between experimental groups. Statistical significance was set at *p* < 0.05. A summary of statistical measures is included in Supplemental Table 1.

## Results

The consequences of an anxious/depressed-like state on episodic, associative or spatial memory were tested in the NORT, the CFC, the MWM, and the BM, respectively.

Long-term glucocorticoid exposure induced physiological changes such as an altered body weight (Figure S1A) (*p* < 0.01) and physical changes including deterioration of coat state (Figure S1B) similarly to chronic stress (Surget et al., 2008). This measure has been described as a reliable and well-validated index of a depressed-like state (Griebel et al., 2002; Santarelli et al., 2003). CORT-treated mice developed an anxiety-like phenotype in the Open Field test, characterized by a decrease in time spent in the center compared to control mice (*p* < 0.05, Figure S1C), but no modification of total ambulatory distance (*p* > 0.1, Figure S1D). We then investigated whether the deterioration of the coat state was linked to changes in grooming behavior. In the Splash test, we observed a decrease in grooming duration after squirting a 10% sucrose solution on the mouse's snout (*p* < 0.01, Figure S1E). Taken together, our results confirm through various behavioral readouts that mice displayed an anxiety/depression-like phenotype induced by an excess of glucocorticoids.

### Chronic corticosterone impaired episodic memory in the novel object recognition test

The NORT procedure was conducted 4 weeks after the start of the corticosterone treatment (see Figures 1, 2A). Control mice showed a novel object preference illustrated by an increase in exploration duration and exploration frequency (Figures 2B,C) of the novel object compared to the familiar one. Although the novel object investigation duration was not higher in CORT-treated mice compared to controls, the number of visits of the novel object was significantly increased in comparison to the familiar object (Figures 2B,C). Taking these data together, both experimental groups showed a novel object preference characterized by an increase in its relative exploration time (*p* < 0.01 vs. the chance level 50% for each group, Figure 2D), with a greater distinction for the novel object in control animals than CORT-treated animals (*p* < 0.01, Figure 2D). Consequently, CORT-treated mice showed a significant decrease in discrimination index compared to control mice (*p* < 0.01, Figure 2E), reflecting a reduction of episodic retention. It is unlikely that this effect was the consequence of a change in objects exploration time (*p* < 0.05, Figure S2A) because locomotor activity did not differ between groups across sessions (*p* > 0.05, Figure S2B).

Then, we assessed whether associative memory was active in CORT-treated mice using an hippocampal-dependent task, the one-trial CFC.

### Altered associative memory after a chronic corticosterone administration in the one-trial contextual fear conditioning

**Figure 3:**
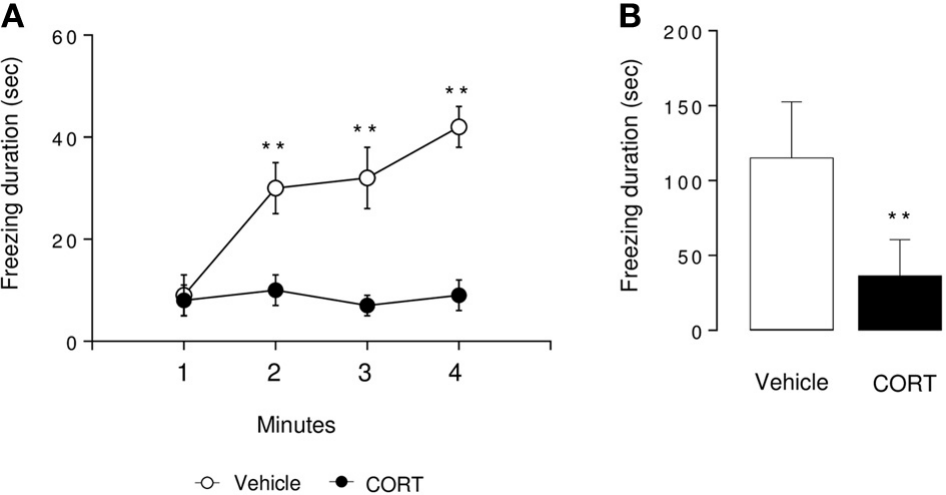
**Associative memory is impaired in CORT-treated mice in one-trial contextual fear conditioning**. Contextual fear conditioning was produced by placing a mouse in the conditioning chamber and delivering one footshock 180 s later. Mice were returned to the conditioning chamber 24 h later to assess for context-elicited freezing. During Day 2, context-elicited freezing was analyzed for each minute of the test (ANOVA with repeated measures) **(A)** and during the whole session **(B)**. Values are mean ± s.e.m., *n* = 5–7 animals per group ^**^p < 0.01 vs. vehicle group.

In the context-elicited fear following training, CORT-treated animals exhibited significantly less freezing than the control animals along the test (*p* < 0.01, Figures 3A,B). These data are in favor of an impairment of the associative memory in CORT-treated animals. As a validation of the experiment, freezing was measured before and after the shock on Day 1. The shock induced an increase in the freezing in both groups of animals (*p* < 0.01, Figure S3). Similarly, no difference occurred between CORT-treated and control animals before or after the shock (*p* > 0.05, Figure S3).

**Figure 4:**
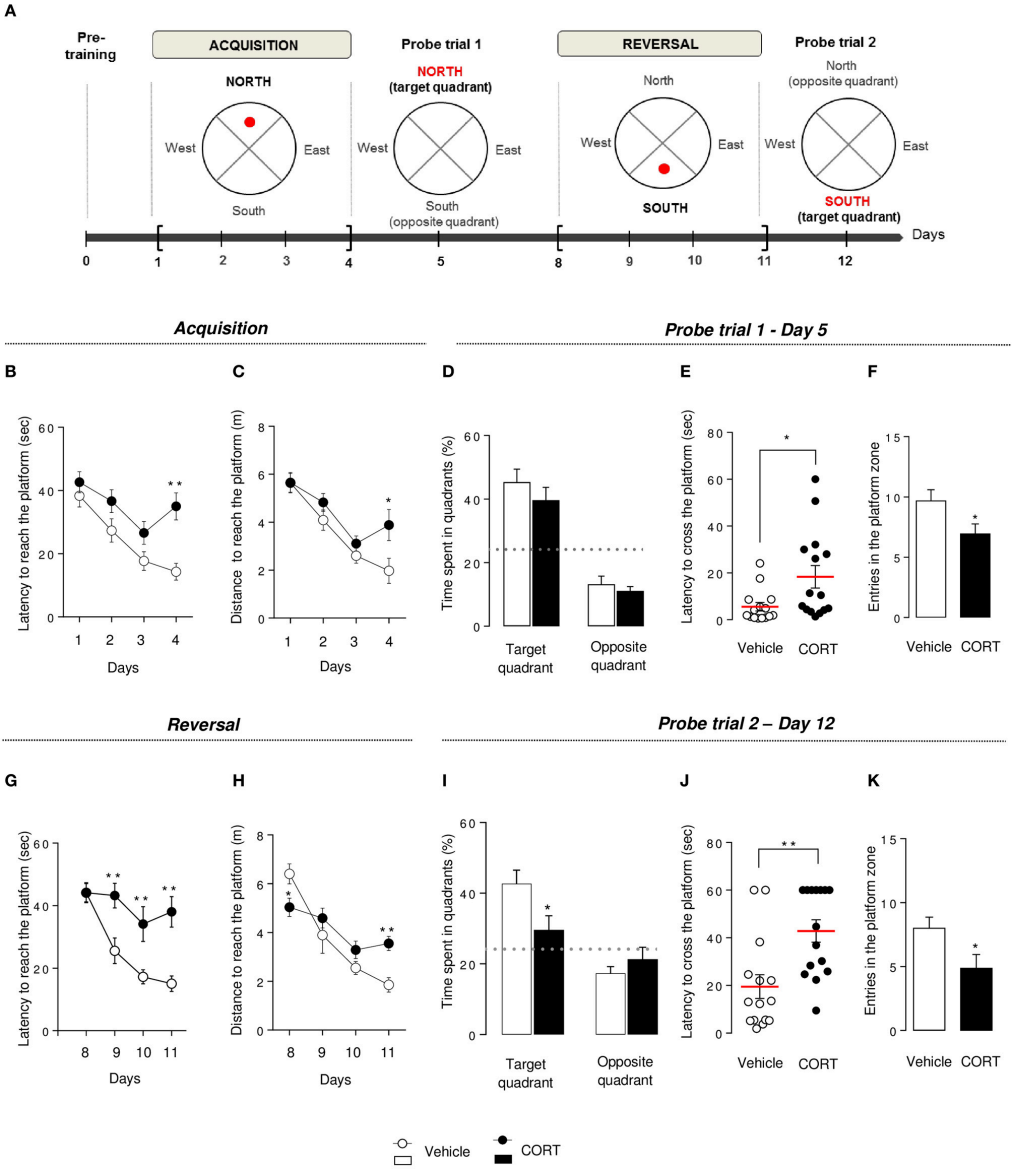
**Chronic corticosterone affects spatial learning performances, short-term memory and cognitive flexibility in the Morris water maze**. Schematic diagram of the MWM experimental time course **(A)**. After a pre-training phase (day 0), the MWM protocol was divided into 2 phases. Each phase includes a 4-day learning period followed by a probe session without the platform. During acquisition, mice learned to locate the platform in the target quadrant (North) then have to locate the platform in the opposite quadrant (South) during reversal. During acquisition, learning was expressed as the latency **(B)** and the total distance traveled **(C)** to reach the hidden platform during training sessions (Day 1–4). Short-term memory was assessed during the probe test in Day 5 by measuring the time spent into the target and the opposite quadrants **(D)**, the latency to first cross the platform zone **(E)** and the number of entries in the platform zone **(F)**. During reversal, learning, and cognitive flexibility were expressed as the latency **(G)** and the total distance traveled **(H)** to reach the hidden platform during training sessions (Day 8–11). Cognitive flexibility was assessed during the probe trial in Day 12 by measuring the time spent into the target and the opposite quadrants **(I)**, the latency to first cross the platform zone **(J)** and the number of entries in the platform zone **(K)**. Values are mean ± s.e.m., *n* = 10–15 animals per group; ^*^p < 0.05; ^**^p < 0.01 vs. vehicle group. The dotted-line indicates the chance level.

**Figure 5:**
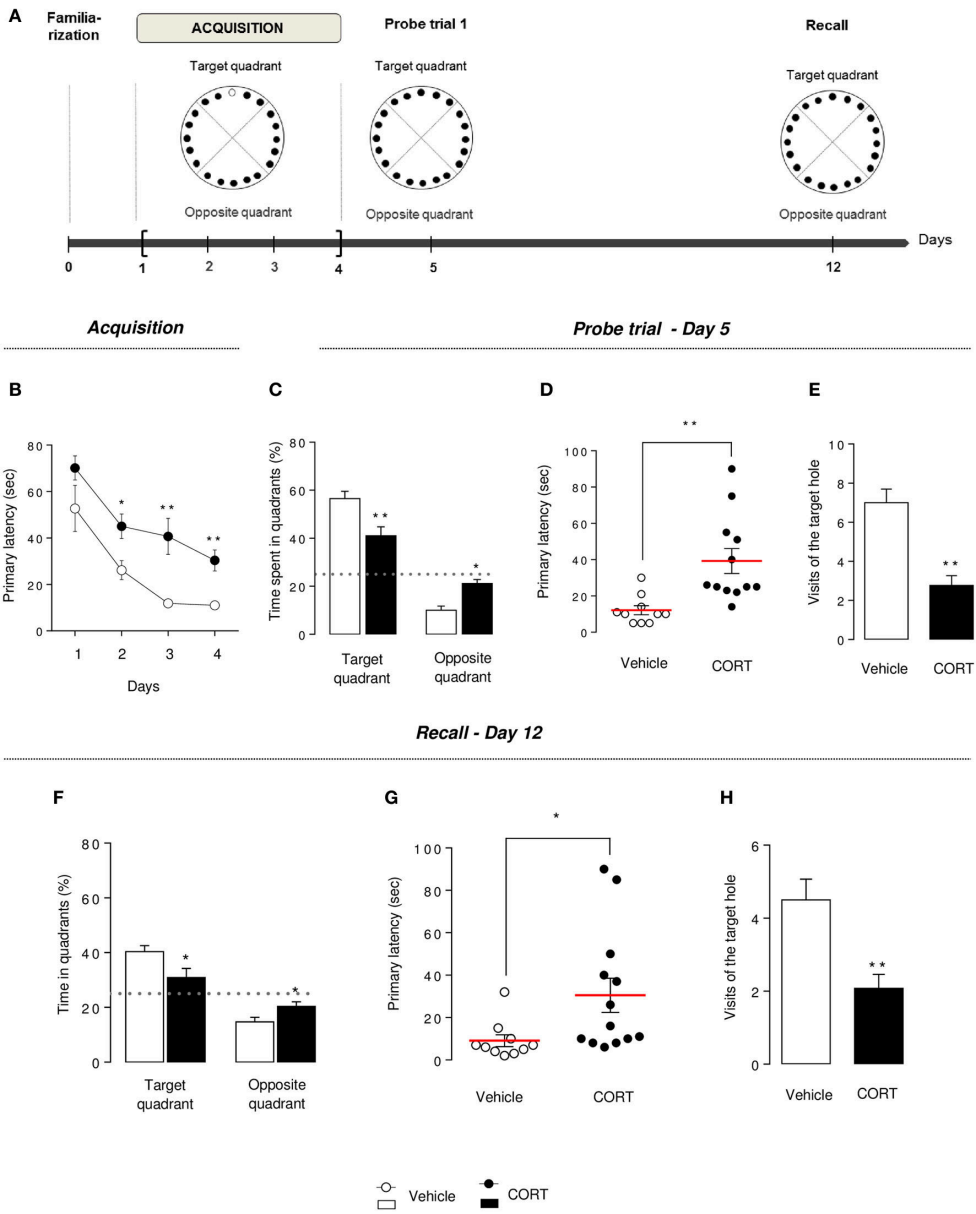
**Chronic corticosterone affects spatial learning performances and memory in the Barnes maze**. Schematic diagram of the of the BM experimental time course **(A)**. Animals were trained during 4 days to learn the location of the target box. A first probe trial estimates short-term memory (Day 5). A second probe trial (recall) estimates long-term memory (Day 12). During acquisition (Day 1–4), learning was monitored by recording primary latency during the training sessions **(B)**. During the first probe trial (Day 5), the target box was removed and the target hole was closed. Short-term memory retention was evaluated by measuring the time spent into the target and the opposite quadrants **(C)**, the primary latency **(D)** and the number of visits in the target hole **(E)**. Mice were not trained from Day 6 to 11. During the recall trial, spatial long-term memory was assessed by measuring the time spent into the target and the opposite quadrants **(F)**, the primary latency **(G)** and the number of visits in the target hole **(H)**. Values are mean ± s.e.m., *n* = 10–13 animals per group; ^*^p < 0.05; ^**^p < 0.01 vs. vehicle group.

Then, to determine the consequences of a chronic CORT treatment on spatial learning and memory performances, mice were submitted the hippocampal-dependent MWM or the BM. Learning was evaluated in both tests through an acquisition phase (Day 1–4) followed by a probe trial (Day 5) to assess short-term retention (Figures 4A, 5A).

### Chronic corticosterone impaired learning, memory and cognitive flexibility in the morris water maze

#### Morris water maze pre-training phase

Motivational behavior to swim was controlled by measuring latency and distance to reach the visible platform during the pre-training phase. The latency for control and CORT-treated mice i.e., 37.5 ± 2.59 and 38.99 ± 2.87 s, respectively, or the distance: 11.53 ± 1.43 and 10.46 ± 1.22 m for control and CORT-treated mice, respectively, did not differ between groups (*t*-test on latency *p* = 0.70; distance *p* = 0.57) indicating no difference in motivational behavior between these groups (Figures S4A,B).

#### Morris water maze acquisition

In the MWM, in contrast to the first 3 days of the acquisition, an increase in latency to reach the hidden platform (*p* < 0.01, Figure 4B) and an increase in path length to reach the platform (*p* < 0.01, Figure 4C) were observed on Day 4 in the CORT-treated animals in comparison to controls. Overall, an anxio/depressed-like state in mice affected spatial learning performance.

#### Morris water maze probe trial 1

Following training sessions, mice were subjected to a probe trial on Day 5 to assess short-term spatial memory. Both groups showed a preference for the target quadrant compared to the chance level (*p* < 0.01, Figure 4D), but CORT-treated mice showed a statistically significant increase in the time to cross the platform for the first time (*p* < 0.05, Figure 4E) and a decrease in the entries in the initial platform target zone (*p* < 0.05, Figure 4F) compared to control animals.

Taken together, these results reveal that CORT treatment affects short-term spatial retention in the MWM.

#### Morris water maze reversal

To test cognitive flexibility, the platform was moved from the target quadrant (North) to the opposite quadrant (South). Mice were then trained during 4 days (Day 8–11) to learn the new location of the hidden platform. Interestingly, from Day 9 to 11, the latency to reach the new location of the platform was significantly increased in CORT-treated mice compared to control group (*p* < 0.01, Figure 4G) without affecting the distance traveled to reach the platform (*p* > 0.1, Figure 4H). However, a significant interaction revealed a decrease in the latency to reach the platform in CORT-treated mice on Day 8 and an increase in this latency on Day 11 (*p* < 0.05 and *p* < 0.01, respectively). We also measured the time spent in the former target quadrant (North) during the 2 first days of the reversal protocol (Day 8–9) (Figure S4C). On Day 8, both groups spent significantly more time in the former target quadrant (*p* < 0.05 vs. the chance level 25%). In contrast, on Day 9, unlike control animals, CORT-treated mice still spent significantly more time in the former target quadrant (*p* < 0.05).

These data suggest that more than an impairment of spatial learning, cognitive flexibility is altered in CORT-treated mice as they failed to adapt their behavior to learn a new platform location.

#### Morris water maze probe trial 2

Following the reversal period, all animals were submitted to a probe trial on Day 12, during which CORT-treated mice failed to use extra cues to find the platform location. Unlike probe trial on Day 5, CORT-treated mice did not show preference for the target quadrant in comparison to control animals (*p* < 0.01, Figure 4I). Furthermore, an increase in the time to cross the platform for the first time (*p* < 0.01, Figures 4J, S4D) associated with a decrease in the number of entries in the new target zone was also observed in our anxio/depression-like mouse model (*p* < 0.01, Figure 4K).

### Chronic corticosterone impaired learning, memory and long term retention in the barnes maze

The BM presents similarities to the MWM, assessing spatial learning performance, but without strong aversive stimuli.

#### Barnes maze acquisition

Previous spatial learning impairments observed in the MWM were confirmed in the BM. Indeed, an overall decrease in the primary latency (latency to first reach the target hole) was observed during the 4 training days in both groups, but CORT-treated animals segregate from control animals, starting at Day 2, with an increase in the time to identify the target hole (*p* < 0.01, Figure 5B), an increase in the time to escape into the target box (*p* < 0.01, Figure S5A) and a greater number of errors committed (*p* < 0.01, Figures S5B,C).

These data suggest that spatial learning performances are altered by a chronic CORT treatment in this spatial test.

#### Barnes maze probe trial

On Day 5, short-term spatial memory was evaluated by a probe trial. CORT-treated mice spent significantly less time in the target quadrant and more time in the opposite quadrant compared to vehicle animals (*p* < 0.01, Figure 5C). Similarly to what we observed in the MWM, an impairment of short-term memory was observed in the BM, with an increase in primary latency, a decrease in the number of visits in the target hole and an increase in primary errors (*p* < 0.01, Figures 5D,E, S5D). The analysis of the distribution pattern of visits finally confirmed that CORT-treated mice displayed lower spatial memory performances in this test compared to controls (*p* < 0.01, Figure S5E).

#### Barnes maze recall probe trial

Long-term memory was evaluated in CORT-treated and control mice, a week after the acquisition phase, in similar conditions than the first probe trial.

Unlike controls, CORT-treated mice were not able to find the location of the target hole. Indeed, the time spent in the target quadrant was significantly lower than controls (*p* < 0.01, Figure 5F) and not different from the chance level 25% (*p* > 0.1). A significant increase in primary latency and a significant decrease in the number of visits of the target hole (*p* < 0.05 and *p* < 0.01, respectively, Figures 5G,H) support that CORT-treated mice long-term retention could be affected, despite their acquisition learning difficulties previously demonstrated (Figure 5B).

## Discussion

Our study showed that in a neuroendocrine-based model of depressive-like behavior, not only emotion-related behavior is impacted, but chronic CORT also has a detrimental effect on cognitive performances, including short-term episodic memory, spatial reference learning and memory and associative memory. Indeed, these series of experiments yielded two main results: mice with an anxious-depressed-like phenotype showed (1) a deficit in learning and cognitive flexibility, (2) an impairment of short- and long-term memory.

### Impaired visuo-spatial learning and cognitive flexibility in anxious-depressed-like mice

#### Visuo-spatial learning is impaired in anxious-depressed-like mice

Our study demonstrates that chronic administration of CORT in mice has a dramatic influence on learning in visuo-spatial paradigms such as the BM and the MWM. As shown in our prior studies (David et al., 2009; Mendez-David et al., 2014), no changes in the locomotor activity could account for this effect, as the total ambulatory distance swam in the MWM was similar between groups. The finding that chronic CORT administration causes spatial learning deficits is consistent with previous reports in rats (Bodnoff et al., 1995; McLay et al., 1998; Coburn-Litvak et al., 2003).

Associative learning was also affected after chronic CORT administration. Twenty-four hours after a first exposure to the stressor, CORT-treated animals froze less than controls in the CFC. The learning that underlies this test typically includes two distinct processes: (1) the acquisition of a mental representation of the context and (2) an association between the context representation and the unconditioned stimulus representation (Rudy et al., 2004). CFC with a single context-shock pairing is an adult hippocampal neurogenesis-dependent task, sensitive to the X-irradiation-induced blockade of neurogenesis (Denny et al., 2012). Our findings suggest that CORT-induced deficit in context conditioning may be related to a decrease in cell proliferation of progenitor cells in the dentate gyrus in adult hippocampus that may impede the acquisition of the context representation. Indeed, previous works demonstrated that chronic CORT exposure induces similar effects than a chronic stress on cell proliferation, decreasing the number of BrdU-positive cells in the dentate gyrus of the adult mouse hippocampus (Surget et al., 2008; David et al., 2009).

Other hippocampal subregions may participate to the deleterious effects of chronic CORT. For instance, CORT-treated animals also showed decrease in hippocampal CA3 branch points and total dendritic length in the apical tree that would be causally related with the learning impairment (Bisagno et al., 2000). This effect is shared with those of chronic stress procedures, which have been repeatedly found to produce an atrophy of apical dendrites of CA3 hippocampal pyramidal neurons (Watanabe et al., 1992; Magarinos and McEwen, 1995) as well as learning impairments in hippocampus-dependent tasks.

#### Cognitive flexibility is altered in anxious-depressed-like mice

Cognitive flexibility is an important executive function involving the ability to change a previously learned behavior in response to changes in environmental feedback. Rodents exposed to chronic stress also show reduced cognitive flexibility in attentional set-shifting and reversal learning tasks (Cerqueira et al., 2005, 2007; Bondi et al., 2008), an effect that can be alleviated after acute or subchronic antidepressant treatments (Bondi et al., 2008; Nikiforuk and Popik, 2011; Naegeli et al., 2013). In the MWM, we found similar evidence of loss of cognitive flexibility in our model. Indeed, CORT-treated animals failed to adapt their behavior to learn the new location of the platform, after switching it from the target to the opposite quadrant. Interestingly, in a mouse model of constitutively suppressed adult neurogenesis, the Cyclin D2 knockout mice, impairment in re-learning in the MWM was observed (Garthe et al., 2014). Moreover, re-learning the platform position in an already known general context after goal reversal could requires pattern separation (Wiskott et al., 2006), a process that can be stimulated with increased neurogenesis (Sahay et al., 2011). While this hypothesis was not tested here, we can hypothesize that reduced cognitive flexibility in CORT-treated animals may be linked to changes in adult hippocampal neurogenesis observed in this model.

### Impaired short-term and long-term memory in anxious-depressed-like mice

There has been a growing awareness that mood disorders are associated with distinct patterns of cognitive impairments (Clark, 2009; Gotlib and Joormann, 2010). In this study, episodic (NORT), associative (CFC) and visuo-spatial (MWM and BM) memory were assessed in our model of anxiety-depression in mice.

HPA axis hyperactivation leads to negative effects on memory processes (Song et al., 2006; Aisa et al., 2007; Maccari and Morley-Fletcher, 2007). Specifically, a study demonstrated that mice submitted to learned helplessness paradigm or chronic mild stress procedures showed poor water maze performances (Song et al., 2006). Here, we observed that chronic CORT administration impaired spatial cognitive retention in both mazes. Moreover, because the BM evaluates cognitive function with minimal stress to the animal (vs. the MWM), we confirm that poor performances after chronic glucocorticoid treatment in both mazes are the result of cognitive impairment, rather than a possible effect of stress. Memory retention was also investigated in CORT-treated mice using the NORT. This test strongly relies on visual recognition memory and is based on rodent's exploratory behavior and spontaneous preference for novel objects (Ennaceur and Delacour, 1988). Notably, this task has been a model of short-term episodic memory as mice are able to recognize the familiar object up to a few hours post-training (Bertaina-Anglade et al., 2006). In our study, CORT-treated mice had no difficulties to distinguish between the two objects, but showed an important alteration on short-term memory characterized by a lower discrimination index compared to control mice. Non-spatial NORT is commonly described as a hippocampal-independent task. However, a recent report proposed that the hippocampus was required in this episodic short-term memory test (Cohen et al., 2013), thus raising the hypothesis that lower discrimination index of CORT-treated mice could be attributable to morphologic molecular and cellular changes in brain processes, especially in neurogenesis adaptation. Current studies investigating the relationship between non-spatial NORT and adult hippocampal neurogenesis led to conflicting findings: adult hippocampal neurogenesis alteration is linked either negatively (Jessberger et al., 2009; Cohen et al., 2013) or positively (Denny et al., 2012; Oury et al., 2013) to the novel object discrimination. It seems, at least that the functional integrity of the dentate gyrus is involved in the non-spatial NORT.

We also investigated long-term memory in the BM 1 week after the first probe trial, in similar conditions. In this spatial test, learning behavioral profile in CORT-treated mice makes difficult to interpret the long-term memory assessment. Although acquisition learning and memory were affected in CORT-treated mice, they still succeeded to learn the task. Comparing performances on Day 5 vs. 12 in CORT-treated mice, we showed that these animals lost their ability to locate the target hole a week after the last trial. This finding is in a favor of a change in long-term retention induced by the chronic CORT treatment. Similarities in long-term behavioral changes were observed in a study performed by El Hage et al. (2006), where mice exposed to a unique traumatic stress showed spatial disabilities in the radial maze that persisted beyond a long period, leading to long-term impaired memory.

### Translational applicability of our results

In humans, common physiological mechanisms involving hypothalamo-pituitary-adrenal (HPA) dysfunctions link stress-induced mood disorders and cognitive impairments. It has been shown that enhanced HPA axis activity induced adverse effects on cognitive performances in MDD subjects (Gomez et al., 2009; Hinkelmann et al., 2009). Substantial evidence shows that cognitive symptoms affect a large subset of patients with unipolar depression (for review Marazziti et al., 2010; Trivedi and Greer, 2014). Specifically, the characteristic cognitive profile includes impairments in pattern recognition memory, processing speed, visuo-spatial memory or executive function (McDermott and Ebmeier, 2009). Visuo-spatial learning deficits are well-documented through various automated neuropsychological test battery (Austin et al., 2001; Porter et al., 2003; Egerhazi et al., 2013). In a recent meta-analysis in early MDD, Lee et al. (2012) employed data from 644 patients from 13 different studies, and showed that patients with a first major depressive episode had significant visual learning impairments compared with healthy controls. Similarly, a clinical study assessing neuropsychological functioning showed that major depressive episode patients displayed spatial working memory alterations (Bourke et al., 2012). Few studies aimed at evaluate the cognitive flexibility in unipolar subjects. However, several studies showed that major depression can impair cognitive flexibility (Degl'innocenti et al., 1998; Deveney and Deldin, 2006; Murphy et al., 2012), an effect that could persist even after MDD remission (Hasselbalch et al., 2012).

### Study limitations

The mouse CORT model is a chronic exposure method optimized for use in modeling the persistent anxiety/depression-like state in rodents. Allowing multiple behavioral tests in the same animals, the CORT procedure is an etiologically relevant model of depression that is easily replicable between and within laboratories (Gourley and Taylor, 2009; Mendez-David et al., 2014). However, it does not fully replicate the core pathology of MDD, as animals in this model are not facing environmental stressors, or the greater female susceptibility observed in the disease (Guilloux et al., 2011). Although, it benefits from its reliability and repeatability compared to standard models of depression. Indeed, learned helplessness or chronic mild stress (CMS) procedures are hampered by protocol variability in rodents (Nestler et al., 2002), probably leading to the low reports of co-occurrence of anxiety and depression-like behaviors as well as learning-memory impairments in such a model (Gomez et al., 2013; Haridas et al., 2013).

## Conclusion

Our results highlight that altered emotional phenotype after 4 weeks of chronic CORT treatment induced a cognitive deficit that affects all aspects of learning and memory, especially episodic, associative and visuo-spatial systems in mice. Because cognitive symptoms have a substantial impact on functional recovery and disability associated with depression, therapies are needed to improve or preserve cognition. Future research in this area should evaluate potential cognitive properties of antidepressant in mice under stressful conditions. Considering the controversial results in the literature about beneficial (Song et al., 2006; Couto et al., 2012) or detrimental (Gumuslu et al., 2013) effects of chronic monoaminergic antidepressant drug treatment in cognition, it will be worth to investigate whether chronic antidepressant administration ameliorates cognitive performance in the chronic CORT model. The strong reliability of this animal model of anxiety/depression (see Mendez-David et al., 2013b, for review) will certainly allow the dissection of the mechanisms linking depression, cognitive impairments and antidepressant-treatment response.

## Author contributions

Flavie Darcet, Jean-Philippe Guilloux, and Denis J. David designed research; Flavie Darcet, Indira Mendez-David performed research. Flavie Darcet, Indira Mendez-David, Laurent Tritschler, Alain M. Gardier, Jean-Philippe Guilloux and Denis J. David analyzed data and wrote the manuscript. Flavie Darcet, Indira Mendez-David, Laurent Tritschler, Alain M. Gardier, Jean-Philippe Guilloux and Denis J. David contributed to the preparation of the manuscript.

### Conflict of interest statement

The authors declare that the research was conducted in the absence of any commercial or financial relationships that could be construed as a potential conflict of interest.

## References

[B1] Aisa B. Tordera R. Lasheras B. Del Rio J. Ramirez M. J. (2007). Cognitive impairment associated to HPA axis hyperactivity after maternal separation in rats. Psychoneuroendocrinology 32, 256–266 10.1016/j.psyneuen.2006.12.013 17307298

[B2] Austin M. P. Mitchell P. Goodwin G. M. (2001). Cognitive deficits in depression: possible implications for functional neuropathology. Br. J. Psychiatry 178, 200–206 10.1192/bjp.178.3.200 11230029

[B3] Bertaina-Anglade V. Enjuanes E. Morillon D. Drieu La Rochelle C. (2006). The object recognition task in rats and mice: a simple and rapid model in safety pharmacology to detect amnesic properties of a new chemical entity. J. Pharmacol. Toxicol. Methods 54, 99–105 10.1016/j.vascn.2006.04.001 16750402

[B4] Bisagno V. Ferrini M. Rios H. Zieher L. M. Wikinski S. I. (2000). Chronic corticosterone impairs inhibitory avoidance in rats: possible link with atrophy of hippocampal CA3 neurons. Pharmacol. Biochem. Behav. 66, 235–240 10.1016/S0091-3057(00)00265-3 10880674

[B5] Bodnoff S. R. Humphreys A. G. Lehman J. C. Diamond D. M. Rose G. M. Meaney M. J. (1995). Enduring effects of chronic corticosterone treatment on spatial learning, synaptic plasticity, and hippocampal neuropathology in young and mid-aged rats. J. Neurosci. 15, 61–69 7823152 10.1523/JNEUROSCI.15-01-00061.1995 PMC6578287

[B6] Bondi C. O. Rodriguez G. Gould G. G. Frazer A. Morilak D. A. (2008). Chronic unpredictable stress induces a cognitive deficit and anxiety-like behavior in rats that is prevented by chronic antidepressant drug treatment. Neuropsychopharmacology 33, 320–331 10.1038/sj.npp.1301410 17406647

[B7] Bourke C. Porter R. J. Carter J. D. McIntosh V. V. Jordan J. Bell C. (2012). Comparison of neuropsychological functioning and emotional processing in major depression and social anxiety disorder subjects, and matched healthy controls. Aust. N.Z. J. Psychiatry 46, 972–981 10.1177/0004867412451502 22711880

[B8] Cerqueira J. J. Mailliet F. Almeida O. F. Jay T. M. Sousa N. (2007). The prefrontal cortex as a key target of the maladaptive response to stress. J. Neurosci. 27, 2781–2787 10.1523/JNEUROSCI.4372-06.2007 17360899 PMC6672565

[B9] Cerqueira J. J. Pego J. M. Taipa R. Bessa J. M. Almeida O. F. Sousa N. (2005). Morphological correlates of corticosteroid-induced changes in prefrontal cortex-dependent behaviors. J. Neurosci. 25, 7792–7800 10.1523/JNEUROSCI.1598-05.2005 16120780 PMC6725252

[B10] Clark D. A. (2009). Cognitive behavioral therapy for anxiety and depression: possibilities and limitations of a transdiagnostic perspective. Cogn. Behav. Ther. 38 Suppl. 1, 29–34 10.1080/16506070902980745 22946137

[B11] Coburn-Litvak P. S. Pothakos K. Tata D. A. McCloskey D. P. Anderson B. J. (2003). Chronic administration of corticosterone impairs spatial reference memory before spatial working memory in rats. Neurobiol. Learn. Mem. 80, 11–23 10.1016/S1074-7427(03)00019-4 12737930

[B12] Cohen S. J. Munchow A. H. Rios L. M. Zhang G. Asgeirsdottir H. N. Stackman R. W. Jr. (2013). The rodent hippocampus is essential for nonspatial object memory. Curr. Biol. 23, 1685–1690 10.1016/j.cub.2013.07.002 23954431 PMC3775586

[B13] Couto F. S. Batalha V. L. Valadas J. S. Data-Franca J. Ribeiro J. A. Lopes L. V. (2012). Escitalopram improves memory deficits induced by maternal separation in the rat. Eur. J. Pharmacol. 695, 71–75 10.1016/j.ejphar.2012.08.020 22981666

[B14] David D. J. Samuels B. A. Rainer Q. Wang J. W. Marsteller D. Mendez I. (2009). Neurogenesis-dependent and -independent effects of fluoxetine in an animal model of anxiety/depression. Neuron 62, 479–493 10.1016/j.neuron.2009.04.017 19477151 PMC2759281

[B15] Degl'innocenti A. Agren H. Backman L. (1998). Executive deficits in major depression. Acta Psychiatr. Scand. 97, 182–188 10.1111/j.1600-0447.1998.tb09985.x 9543305

[B16] Denny C. A. Burghardt N. S. Schachter D. M. Hen R. Drew M. R. (2012). 4- to 6-week-old adult-born hippocampal neurons influence novelty-evoked exploration and contextual fear conditioning. Hippocampus 22, 1188–1201 10.1002/hipo.20964 21739523 PMC3193906

[B17] Deveney C. M. Deldin P. J. (2006). A preliminary investigation of cognitive flexibility for emotional information in major depressive disorder and non-psychiatric controls. Emotion 6, 429–437 10.1037/1528-3542.6.3.429 16938084

[B18] Drew M. R. Denny C. A. Hen R. (2010). Arrest of adult hippocampal neurogenesis in mice impairs single- but not multiple-trial contextual fear conditioning. Behav. Neurosci. 124, 446–454 10.1037/a0020081 20695644 PMC2925248

[B19] Egerhazi A. Balla P. Ritzl A. Varga Z. Frecska E. Berecz R. (2013). Automated neuropsychological test battery in depression – preliminary data. Neuropsychopharmacol. Hung. 15, 5–11 23542754

[B20] El Hage W. Griebel G. Belzung C. (2006). Long-term impaired memory following predatory stress in mice. Physiol. Behav. 87, 45–50 10.1016/j.physbeh.2005.08.039 16182325

[B21] Elizalde N. Gil-Bea F. J. Ramirez M. J. Aisa B. Lasheras B. Del Rio J. (2008). Long-lasting behavioral effects and recognition memory deficit induced by chronic mild stress in mice: effect of antidepressant treatment. Psychopharmacology (Berl.) 199, 1–14 10.1007/s00213-007-1035-1 18470507

[B22] Ennaceur A. Delacour J. (1988). A new one-trial test for neurobiological studies of memory in rats. 1: behavioral data. Behav. Brain Res. 31, 47–59 10.1016/0166-4328(88)90157-X 3228475

[B23] Fava M. Graves L. M. Benazzi F. Scalia M. J. Iosifescu D. V. Alpert J. E. (2006). A cross-sectional study of the prevalence of cognitive and physical symptoms during long-term antidepressant treatment. J. Clin. Psychiatry 67, 1754–1759 10.4088/JCP.v67n1113 17196056

[B24] Fossati P. Coyette F. Ergis A. M. Allilaire J. F. (2002). Influence of age and executive functioning on verbal memory of inpatients with depression. J. Affect. Disord. 68, 261–271 10.1016/S0165-0327(00)00362-1 12063154

[B25] Garcia R. Spennato G. Nilsson-Todd L. Moreau J. L. Deschaux O. (2008). Hippocampal low-frequency stimulation and chronic mild stress similarly disrupt fear extinction memory in rats. Neurobiol. Learn. Mem. 89, 560–566 10.1016/j.nlm.2007.10.005 18039585

[B26] Garthe A. Huang Z. Kaczmarek L. Filipkowski R. K. Kempermann G. (2014). Not all water mazes are created equal: cyclin D2 knockout mice with constitutively suppressed adult hippocampal neurogenesis do show specific spatial learning deficits. Genes Brain Behav. 13, 357–364 10.1111/gbb.12130 24602283 PMC4314690

[B27] Gomez J. L. Lewis M. J. Sebastian V. Serrano P. Luine V. N. (2013). Alcohol administration blocks stress-induced impairments in memory and anxiety, and alters hippocampal neurotransmitter receptor expression in male rats. Horm. Behav. 63, 659–666 10.1016/j.yhbeh.2013.01.007 23376488 PMC3646638

[B28] Gomez R. G. Posener J. A. Keller J. Debattista C. Solvason B. Schatzberg A. F. (2009). Effects of major depression diagnosis and cortisol levels on indices of neurocognitive function. Psychoneuroendocrinology 34, 1012–1018 10.1016/j.psyneuen.2009.01.017 19261389

[B29] Gotlib I. H. Joormann J. (2010). Cognition and depression: current status and future directions. Annu. Rev. Clin. Psychol. 6, 285–312 10.1146/annurev.clinpsy.121208.131305 20192795 PMC2845726

[B30] Gourley S. L. Taylor J. R. (2009). Recapitulation and reversal of a persistent depression-like syndrome in rodents. Curr. Protoc. Neurosci. Chapter 9, Unit 9 32. 10.1002/0471142301.ns0932s49 19802817 PMC2774936

[B31] Griebel G. Simiand J. Serradeil-Le Gal C. Wagnon J. Pascal M. Scatton B. (2002). Anxiolytic- and antidepressant-like effects of the non-peptide vasopressin V1b receptor antagonist, SSR149415, suggest an innovative approach for the treatment of stress-related disorders. Proc. Natl. Acad. Sci. U.S.A. 99, 6370–6375 10.1073/pnas.092012099 11959912 PMC122955

[B32] Guilloux J. P. Seney M. Edgar N. Sibille E. (2011). Integrated behavioral z-scoring increases the sensitivity and reliability of behavioral phenotyping in mice: relevance to emotionality and sex. J. Neurosci. Methods 197, 21–31 10.1016/j.jneumeth.2011.01.019 21277897 PMC3086134

[B33] Gumuslu E. Mutlu O. Sunnetci D. Ulak G. Celikyurt I. K. Cine N. (2013). The effects of tianeptine, olanzapine and fluoxetine on the cognitive behaviors of unpredictable chronic mild stress-exposed mice. Drug Res. (Stuttg). 63, 532–539 10.1055/s-0033-1347237 23780498

[B34] Hammar A. Ardal G. (2009). Cognitive functioning in major depression–a summary. Front. Hum. Neurosci. 3:26 10.3389/neuro.09.026.2009 19826496 PMC2759342

[B35] Hammar A. Lund A. Hugdahl K. (2003). Long-lasting cognitive impairment in unipolar major depression: a 6-month follow-up study. Psychiatry Res. 118, 189–196 10.1016/S0165-1781(03)00075-1 12798984

[B36] Haridas S. Kumar M. Manda K. (2013). Melatonin ameliorates chronic mild stress induced behavioral dysfunctions in mice. Physiol. Behav. 119, 201–207 10.1016/j.physbeh.2013.06.015 23810991

[B37] Hasselbalch B. J. Knorr U. Hasselbalch S. G. Gade A. Kessing L. V. (2012). Cognitive deficits in the remitted state of unipolar depressive disorder. Neuropsychology 26, 642–651 10.1037/a0029301 22823136

[B38] Hinkelmann K. Moritz S. Botzenhardt J. Riedesel K. Wiedemann K. Kellner M. (2009). Cognitive impairment in major depression: association with salivary cortisol. Biol. Psychiatry 66, 879–885 10.1016/j.biopsych.2009.06.023 19709646

[B39] Jessberger S. Clark R. E. Broadbent N. J. Clemenson G. D. Jr. Consiglio A. Lie D. C. (2009). Dentate gyrus-specific knockdown of adult neurogenesis impairs spatial and object recognition memory in adult rats. Learn. Mem. 16, 147–154 10.1101/lm.1172609 19181621 PMC2661246

[B40] Lampe I. K. Sitskoorn M. M. Heeren T. J. (2004). Effects of recurrent major depressive disorder on behavior and cognitive function in female depressed patients. Psychiatry Res. 125, 73–79 10.1016/j.psychres.2003.12.004 15006430

[B41] Landro N. I. Stiles T. C. Sletvold H. (2001). Neuropsychological function in nonpsychotic unipolar major depression. Neuropsychiatry Neuropsychol. Behav. Neurol. 14, 233–240 11725217

[B42] Lee R. S. Hermens D. F. Porter M. A. Redoblado-Hodge M. A. (2012). A meta-analysis of cognitive deficits in first-episode major depressive disorder. J. Affect. Disord. 140, 113–124 10.1016/j.jad.2011.10.023 22088608

[B43] Maccari S. Morley-Fletcher S. (2007). Effects of prenatal restraint stress on the hypothalamus-pituitary-adrenal axis and related behavioural and neurobiological alterations. Psychoneuroendocrinology 32 Suppl. 1, S10–S15 10.1016/j.psyneuen.2007.06.005 17651905

[B44] Magarinos A. M. McEwen B. S. (1995). Stress-induced atrophy of apical dendrites of hippocampal CA3c neurons: involvement of glucocorticoid secretion and excitatory amino acid receptors. Neuroscience 69, 89–98 10.1016/0306-4522(95)00259-L 8637636

[B45] Marazziti D. Consoli G. Picchetti M. Carlini M. Faravelli L. (2010). Cognitive impairment in major depression. Eur. J. Pharmacol. 626, 83–86 10.1016/j.ejphar.2009.08.046 19835870

[B46] McDermott L. M. Ebmeier K. P. (2009). A meta-analysis of depression severity and cognitive function. J. Affect. Disord. 119, 1–8 10.1016/j.jad.2009.04.022 19428120

[B47] McLay R. N. Freeman S. M. Zadina J. E. (1998). Chronic corticosterone impairs memory performance in the Barnes maze. Physiol. Behav. 63, 933–937 10.1016/S0031-9384(97)00529-5 9618019

[B48] Mendez-David I. David D. J. Darcet F. Wu M. V. Kerdine-Romer S. Gardier A. M. (2014). Rapid anxiolytic effects of a 5-HT receptor agonist are mediated by a neurogenesis-independent mechanism. Neuropsychopharmacology. 39, 1366–1378 10.1038/npp.2013.332 24287720 PMC3988540

[B49] Mendez-David I. Hen R. Gardier A. M. David D. J. (2013b). Adult hippocampal neurogenesis: an actor in the antidepressant-like action. Ann. Pharm. Fr. 71, 143–149 10.1016/j.pharma.2013.02.006 23622692

[B50] Millan M. J. Agid Y. Brune M. Bullmore E. T. Carter C. S. Clayton N. S. (2012). Cognitive dysfunction in psychiatric disorders: characteristics, causes and the quest for improved therapy. Nat. Rev. Drug Discov. 11, 141–168 10.1038/nrd3628 22293568

[B51] Murphy F. C. Michael A. Sahakian B. J. (2012). Emotion modulates cognitive flexibility in patients with major depression. Psychol. Med. 42, 1373–1382 10.1017/S0033291711002418 22067530

[B52] Murrough J. W. Iacoviello B. Neumeister A. Charney D. S. Iosifescu D. V. (2011). Cognitive dysfunction in depression: neurocircuitry and new therapeutic strategies. Neurobiol. Learn. Mem. 96, 553–563 10.1016/j.nlm.2011.06.006 21704176

[B53] Naegeli K. J. O'connor J. A. Banerjee P. Morilak D. A. (2013). Effects of milnacipran on cognitive flexibility following chronic stress in rats. Eur. J. Pharmacol. 703, 62–66 10.1016/j.ejphar.2013.02.006 23422875

[B54] Naismith S. L. Hickie I. B. Turner K. Little C. L. Winter V. Ward P. B. (2003). Neuropsychological performance in patients with depression is associated with clinical, etiological and genetic risk factors. J. Clin. Exp. Neuropsychol. 25, 866–877 10.1076/jcen.25.6.866.16472 13680463

[B55] Nestler E. J. Barrot M. Dileone R. J. Eisch A. J. Gold S. J. Monteggia L. M. (2002). Neurobiology of depression. Neuron 34, 13–25 10.1016/S0896-6273(02)00653-0 11931738

[B56] Nikiforuk A. Popik P. (2011). Long-lasting cognitive deficit induced by stress is alleviated by acute administration of antidepressants. Psychoneuroendocrinology 36, 28–39 10.1016/j.psyneuen.2010.06.001 20580164

[B57] Orsetti M. Colella L. Dellarole A. Canonico P. L. Ghi P. (2007). Modification of spatial recognition memory and object discrimination after chronic administration of haloperidol, amitriptyline, sodium valproate or olanzapine in normal and anhedonic rats. Int. J. Neuropsychopharmacol. 10, 345–357 10.1017/S1461145706006705 16734936

[B58] Oury F. Khrimian L. Denny C. A. Gardin A. Chamouni A. Goeden N. (2013). Maternal and offspring pools of osteocalcin influence brain development and functions. Cell 155, 228–241 10.1016/j.cell.2013.08.042 24074871 PMC3864001

[B59] Patki G. Solanki N. Atrooz F. Allam F. Salim S. (2013). Depression, anxiety-like behavior and memory impairment are associated with increased oxidative stress and inflammation in a rat model of social stress. Brain Res. 1539, 73–86 10.1016/j.brainres.2013.09.033 24096214 PMC4316676

[B60] Porter R. J. Gallagher P. Thompson J. M. Young A. H. (2003). Neurocognitive impairment in drug-free patients with major depressive disorder. Br. J. Psychiatry 182, 214–220 10.1192/bjp.182.3.214 12611784

[B61] Ravnkilde B. Videbech P. Clemmensen K. Egander A. Rasmussen N. A. Rosenberg R. (2002). Cognitive deficits in major depression. Scand. J. Psychol. 43, 239–251 10.1111/1467-9450.00292 12184479

[B62] Richter S. H. Zeuch B. Lankisch K. Gass P. Durstewitz D. Vollmayr B. (2013). Where have I been? Where should I go? Spatial working memory on a radial arm maze in a rat model of depression. PLoS ONE 8:e62458 10.1371/journal.pone.0062458 23614050 PMC3632551

[B63] Rudy J. W. Huff N. C. Matus-Amat P. (2004). Understanding contextual fear conditioning: insights from a two-process model. Neurosci. Biobehav. Rev. 28, 675–685 10.1016/j.neubiorev.2004.09.004 15555677

[B64] Sahay A. Scobie K. N. Hill A. S. O'carroll C. M. Kheirbek M. A. Burghardt N. S. (2011). Increasing adult hippocampal neurogenesis is sufficient to improve pattern separation. Nature 472, 466–470 10.1038/nature09817 21460835 PMC3084370

[B65] Santarelli L. Saxe M. Gross C. Surget A. Battaglia F. Dulawa S. (2003). Requirement of hippocampal neurogenesis for the behavioral effects of antidepressants. Science 301, 805–809 10.1126/science.1083328 12907793

[B66] Song L. Che W. Min-Wei W. Murakami Y. Matsumoto K. (2006). Impairment of the spatial learning and memory induced by learned helplessness and chronic mild stress. Pharmacol. Biochem. Behav. 83, 186–193 10.1016/j.pbb.2006.01.004 16519925

[B67] Sunyer B. Patil S. Höger H. Lubec G. (2007). Barnes maze, a useful task to assess spatial reference memory in the mice. Protocol Exchange 10.1038/nprot.2007.390

[B68] Surget A. Saxe M. Leman S. Ibarguen-Vargas Y. Chalon S. Griebel G. (2008). Drug-dependent requirement of hippocampal neurogenesis in a model of depression and of antidepressant reversal. Biol. Psychiatry 64, 293–301 10.1016/j.biopsych.2008.02.022 18406399

[B69] Trivedi M. H. Greer T. L. (2014). Cognitive dysfunction in unipolar depression: implications for treatment. J. Affect. Disord. 152–154, 19–27 10.1016/j.jad.2013.09.012 24215896

[B70] Vythilingam M. Vermetten E. Anderson G. M. Luckenbaugh D. Anderson E. R. Snow J. (2004). Hippocampal volume, memory, and cortisol status in major depressive disorder: effects of treatment. Biol. Psychiatry 56, 101–112 10.1016/j.biopsych.2004.04.002 15231442

[B71] Watanabe Y. Gould E. Cameron H. A. Daniels D. C. McEwen B. S. (1992). Phenytoin prevents stress- and corticosterone-induced atrophy of CA3 pyramidal neurons. Hippocampus 2, 431–435 10.1002/hipo.450020410 1308199

[B72] WHO (2008). World Health Organization - The Global Burden of Disease - 2004 Update. Geneva: WHO Library

[B73] Wiskott L. Rasch M. J. Kempermann G. (2006). A functional hypothesis for adult hippocampal neurogenesis: avoidance of catastrophic interference in the dentate gyrus. Hippocampus 16, 329–343 10.1002/hipo.20167 16435309

