# Supplementary material for: Learning and memory impairments in a neuroendocrine mouse model of anxiety/depression
Source: Front Behav Neurosci. 2014 May 1;8:136. doi: 10.3389/fnbeh.2014.00136 (PMC4013464; doi:10.3389/fnbeh.2014.00136)
Supplement: Supplementary file 1 [file DataSheet1.PDF]

Learning and memory impairments in a neuroendocrine mouse model of anxiety/depression

Flavie Darcet, Indira Mendez-David, Laurent Tritschler, Alain M. Gardier, Jean-Philippe Guilloux*, Denis J. David*,§

Université Paris-Sud, EA3544, Faculté de Pharmacie,
5 rue Jean-Baptiste Clément, Châtenay-Malabry, France

*Last co-authorship.
§ To whom correspondence should be addressed.

Dr Denis DAVID
Univ Paris-Sud
Fac Pharmacie
5, rue J-B Clément, Tour D1, 2e etage
EA 3544 "Pharmacologie des troubles anxio-dépressifs et Neurogenèse"
F-92296 Chatenay-Malabry cedex
tel: (33) 1 46 83 59 68
Fax: (33) 1 46 83 53 55
E-mail: denis.david@u-psud.fr

SUPPLEMENTAL FIGURES

Supplemental Figure 1: Effects of chronic corticosterone treatment on the anxious/depressed-like phenotype

Effects of a chronic CORT treatment on the anxious/depressed-like phenotype (**A**) Weekly measures of mouse body weight during all the protocol showed a greater gain weight in CORT-treated mice compared to control mice. (**B**) Chronic CORT treatment induced a significant alteration of the fur coat state, reflected by an increasing coat state score from week 2 to week 6. Anxiety, evaluated in the Open Field test, was expressed as (**C-D**) time spent in the center, in seconds, for the entire session (**C**) and also as ambulatory distance in the center, in meters, for the entire session (**D**). Depressive-like phenotype assessed in the Splash test was expressed as mean duration of grooming after receiving a 10% sucrose solution on the snout. (**E**) Values are mean ± SEM (n=20 animals per group, Cohort 1) *$p<0.05$; **$p<0.01$ *versus* vehicle group.

Supplemental Figure 2: Effects of chronic corticosterone treatment on exploration duration and locomotor activity in the novel object recognition test.

Effects of chronic CORT treatment on both objects exploration time (**A**) and ambulatory distance (**B**) during training and test sessions. Values are mean ± SEM (n=20 animals per group) **$p<0.01$ *versus* vehicle group.

Supplemental Figure 3: Effects of a chronic corticosterone treatment on freezing duration during shock exposure in the one-trial contextual fear conditioning.

The percentage of freezing was measured before and after the shock during Day 1. A one-way ANOVA with repeated-measures was applied. Values are mean ± SEM (n=5-7 animals per group) **$p<0.01$ *versus* before the shock.

Supplemental Figure 4: Effects of a chronic corticosterone treatment on pre-training and reversal parameters in the Morris water maze.

Latency (**A**) and distance travelled (**B**) to reach the visible platform were recorded during the pre-training phase (Day 0). Relative time spent in the former target quadrant (North) was measured on Day 8 and Day 9 (**C**). A survival curve was performed for latency to first cross the platform during the reversal probe trial in Day 12 (**D**). Values are mean ± SEM (n=15 animals per group) **p<0.01 *versus* vehicle group.

Supplemental Figure 5: Effects of a chronic corticosterone treatment on various learning parameters and short-term memory retention in the Barnes maze.

Total latency (**A**), primary errors (**B**) and total errors (**C**) were recorded in the Barnes maze as learning indicators. During the probe trial on Day 5, the number of primary errors was calculated (**D**) and the number of visits among all holes of the maze were counted to compare patterns of distribution (**E**). Values are mean ± SEM (n=10-13 animals per group) *p<0.05; **p<0.01 *versus* vehicle group.

Supplemental Table 1: complete statistical summary analysis for behavioral data

Behavioral paradigm	Measurement	Statistical Test	Comparison	Statistics	Degrees of freedom	p	Fig.
**Novel object recognition test**	Exploration time	Two-way ANOVA	Factor 1 Treatment	F=10.23	1,76	p<0.01**	**2B**
			Factor 2 Object	F=40.98	1,76	p<0.01**	
			Interaction (F1 X F2)	F=23.18	1,76	p<0.01**	
		PLSD Post-hoc test	CORT *vs* Veh (Novel Object, Familiar Object)			p<0.01**	
	Exploration frequency	Two-way ANOVA	Factor 1 Treatment	F=4.18	1,76	p<0.05*	**2C**
			Factor 2 Object	F=51.12	1,76	p<0.01**	
			Interaction (F1 X F2)	F=12.43	1,76	p<0.01**	
		PLSD Post-hoc test	CORT *vs* Veh (Novel object, Familiar Object)			p<0.01**	
			Familiar *vs* Novel object (Veh)			p<0.01**	
			Familiar *vs* Novel object (CORT)			p<0.05*	
	Exploration time	*t*-test	CORT *vs* Veh	F=48.44	38	p<0.01**	**2D**
		One sample *t*-test	CORT *vs* 50%	t=4.015	19	p<0.01**	
			Veh *vs* 50%	t=10.63	19	p<0.01**	
	Discrimination Index	*t*-test	CORT *vs* Veh	F=48.44	38	p<0.01**	**2E**
	Exploration time across sessions	One-way ANOVA with repeated measures	Factor 1 Treatment	F=15.37	1,114	p<0.01**	**S2A**
			Factor 2 Time	F=9.068	3,114	p<0.01**	
			Interaction (F1 X F2)	F=1.481	3,114	p>0.1	
		PLSD Post-hoc test	CORT *vs* Veh (Session 4)			p<0.01**	
	Total exploration time (Probe test)	*t*-test	CORT *vs* Veh	F=8.23	38	p<0.01**	
	Distance travelled across sessions	One-way ANOVA with repeated measures	Factor 1 Treatment	F=3.425	1,114	p>0.05	**S2B**
			Factor 2 Time	F=47.80	3,114	p<0.01**	
			Interaction (F1 X F2)	F=0.21	3,114	p>0.1	
	Distance travelled (Probe test)	*t*-test	CORT *vs* Veh	F=0.019	38	p>0.5	
**One-trial contextual fear conditioning**	Freezing duration	One-way ANOVA with repeated measures	Factor 1 Treatment	F=19.83	1,30	p<0.01**	**3A**
			Factor 2 Time	F=6.11	3,30	p<0.01**	
			Interaction (F1 X F2)	F=5.24	3,30	p<0.01**	
		PLSD Post-hoc test	CORT *vs* Veh, (minute 2)			p<0.05*	
			CORT *vs* Veh, (minute 3 and 4)			p<0.01**	
	Freezing duration	*t*-test	CORT *vs* Veh	F=19.84	10	p<0.01**	**3B**
	Freezing duration during Day 1	Two-way ANOVA	Factor 1 Treatment	F=0.523	1,20	p>0.5	**S3**
			Factor 2 Time	F=20.35	1,20	p<0.01**	
			Interaction (F1 X F2)	F=0.073	1,20	p>0.1	

**Morris water maze**	Latency to reach the platform (Acquisition)	One-way ANOVA with repeated measures	Factor 1 Treatment	F=10.92	1,84	p<0.01**	**4B**
			Factor 2 Time	F=14.99	3,84	p<0.01**	
			Interaction (F1 X F2)	F=2.67	3,84	p=0.0525	
		PLSD Post-hoc test	CORT *vs* Veh (Day 4)			p<0.01**	
	Total distance (Acquisition)	One-way ANOVA with repeated measures	Factor 1 Treatment	F=5.57	1,84	p<0.05*	**4C**
			Factor 2 Time	F=24.84	1,84	p<0.01**	
			Interaction (F1 X F2)	F=2.24	1,84	p>0.05	
		PLSD Post-hoc test	CORT *vs* Veh (Day 4)			p<0.01**	
	Total distance during probe test-Day 12	*t*-test	CORT *vs* Veh	F=3.385	1,28	p>0.05	
	Time in quadrants (Probe test-Day 5)	Two-way ANOVA	Factor 1 Treatment	F=1.40	1,56	p>0.05	**4D**
			Factor 2 Quadrant	F=22.86	1,56	p<0.01**	
			Interaction (F1 X F2)	F=4.295	1,56	p<0.05*	
		One sample *t*-test	CORT *vs* 25%	t=8.479	14	p<0.01**	
			Veh *vs* 25%	t=8.971	14	p<0.01**	
	Latency to cross the platform (Probe test-Day 5)	*t*-test	CORT *vs* Veh	F=6.55	28	p<0.05*	**4E**
	Entries in the platform zone (Probe test-Day 5)	*t*-test	CORT *vs* Veh	F=4.47	28	p<0.05*	**4F**
	Latency to reach the platform (Reversal)	One-way ANOVA with repeated measures	Factor 1 Treatment	F=11.88	1,84	p<0.01**	**4G**
			Factor 2 Time	F=18.76	3,84	p<0.01**	
			Interaction (F1 X F2)	F=6.32	3,84	p<0.01**	
		PLSD Post-hoc test	CORT *vs* Veh (Day 2 to Day 4)			p<0.01**	
	Total distance (Reversal)	One-way ANOVA with repeated measures	Factor 1 Treatment	F=1.95	1,84	p>0.1	**4H**
			Factor 2 Time	F=34.78	3,84	p<0.01**	
			Interaction (F1 X F2)	F=7.49	3,84	p<0.01**	
		PLSD Post-hoc test	CORT *vs* Veh (Day 8)			p<0.05*	
			CORT *vs* Veh (Day 11)			p<0.01**	
	Total distance during probe test-Day 12	*t*-test	CORT *vs* Veh	F= 3.613	1,28	p>0.05	
	Time in quadrants (Probe test-Day 12)	Two-way ANOVA	Factor 1 Treatment	F=1.76	1,56	p>0.05	**4I**
			Factor 2 Quadrant	F=23.27	1,56	p<0.01**	

			Interaction (F1 X F2)	F=5.97	1,56	p<0.01**	
		PLSD Post-hoc test	CORT *vs* Veh (Target quadrant)			p<0.05*	
		One sample *t*-test	CORT *vs* 25%	t=1.086	14	p>0.2	
			Veh *vs* 25%	t=4.496	14	p<0.01**	
	Latency to cross the platform (Probe test- Day 12)	*t*-test	CORT *vs* Veh	F=11.96	28	p<0.01**	**4J**
	Entries in the platform zone (Probe test- Day 12)	*t*-test	CORT *vs* Veh	F=5.15	28	p<0.05*	**4K**
	Latency to reach the visible platform	*t*-test	CORT *vs* Veh	F=0.386	28	p>0.5	**S4A**
	Distance to reach the visible platform	*t*-test	CORT *vs* Veh	F=0.569	28	p>0.1	**S4B**
	Time spent in former target quadrant (North)	One-way repeated measures ANOVA	Factor 1 Treatment	F=4.963	1,56	p<0.05*	**S4C**
			Factor 2 Time	F=2.946	1,56	p>0.5	
			Interaction (F1 X F2)	F=1.327	1,56	p>0.5	
		PLSD Post-hoc test	CORT *vs* Veh (Day 9)			p<0.05*	
		One sample *t*-test	CORT *vs* 25% (Day 8)	t=2.658	14	p<0.05*	
			Veh *vs* 25% (Day 8)	t=2.301	14	p<0.05*	
			CORT *vs* 25% (Day 9)	t=2.379	14	p<0.05*	
	Latency to cross the platform (Probe test- Day 12)	Mantel-Cox test	Kaplan-Meier Survival analysis			p<0.01**	**S4D**
**Barnes maze**	Primary latency	One-way repeated measures ANOVA	Factor 1 Treatment	F=13.74	1,60	p<0.01**	**5B**
			Factor 2 Time	F=26.69	3,60	p<0.01**	
			Interaction (F1 X F2)	F=0.60	3,60	p>0.6	
		PLSD Post-hoc test	CORT *vs* Veh (Day 2)			p<0.05*	
			CORT *vs* Veh (Day 3, Day 4)			p<0.01**	

	Time in quadrants (Day 5 probe test)	Two-way ANOVA	Factor 1 Treatment	F=0.73	1,42	p>0.05	**5C**
			Factor 2 Quadrant	F=151.0	1,42	p<0.01**	
			Interaction (F1 X F2)	F=19.41	1,42	p<0.01**	
		PLSD Post-hoc test	CORT *vs* Veh (Opposite quadrant)			p<0.05*	
			CORT *vs* Veh (Target quadrant)			p<0.01**	
		One sample *t*-test	CORT *vs* 25%	t=4.79	12	p<0.01**	
			Veh *vs* 25%	t=10.26	9	p<0.01**	
	Primary latency (Day 5 probe trial)	*t*-test	CORT *vs* Veh	F=11.31	21	p<0.01**	**5D**
	Visits of the target hole (Day 5 probe trial)	*t*-test	CORT *vs* Veh	F=25.81	21	p<0.01**	**5E**
	Time in quadrants (Day 12 probe trial)	Two-way ANOVA	Factor 1 Treatment	F=0.57	1,42	p>0.05	**5F**
			Factor 2 Quadrant	F=53.67	1,42	p<0.01**	
			Interaction (F1 X F2)	F=9.43	1,42	p<0.01**	
		PLSD Post-hoc test	CORT *vs* Veh (Target, opposite quadrant)			p<0.05*	
		One sample *t*-test	CORT *vs* 25%	t=1.76	12	p<0.01**	
			Veh *vs* 25%	t=6.81	9	p<0.01**	
	Primary latency (Day 12 probe trial)	*t*-test	CORT *vs* Veh	t=5.05	21	p<0.05*	**5G**
	Visits of the target hole (Day 12 probe trial)	*t*-test	CORT *vs* Veh	t=23.69	21	p<0.01**	**5H**
	Total latency (Acquisition)	One-way ANOVA with repeated measures	Factor 1 Treatment	F=16.21	1,60	p<0.01**	**S5A**
			Factor 2 Time	F=15.81	3,60	p<0.01**	
			Interaction (F1 X F2)	F=3.59	3,60	p<0.05*	
		PLSD Post-hoc test	CORT *vs* Veh (Day 2, Day 3, Day 4)			p<0.01**	
	Primary errors	One-way ANOVA with repeated measures	Factor 1 Treatment	F=46.48	1,60	p<0.01**	**S5B**
			Factor 2 Time	F=3.94	3,60	p<0.05*	
			Interaction (F1 X F2)	F=0.59	3,60	p>0.6	
		PLSD	CORT *vs* Veh (Day 1)			p<0.05*	

		Post-hoc test	CORT *vs* Veh (Day 2, Day 3, Day 4)			p<0.01**	
	Total errors	One-way ANOVA with repeated measures	Factor 1 Treatment	F=22.50	1,60	p<0.01**	S5C
			Factor 2 Time	F=2.96	3,60	p<0.05*	
			Interaction (F1 X F2)	F=5.00	3,60	p<0.01**	
		PLSD Post-hoc test	CORT *vs* Veh (Day 2, Day 3, Day 4)			p<0.01**	
	Primary errors (Day 5 probe trial)	*t*-test	CORT *vs* Veh	F=8.95	21	p<0.01**	S5D
	Visits distribution	Two-way ANOVA	Factor 1 Treatment	F=25.9	1,42	p<0.01**	S5E
			Factor 2 Hole	F=32.46	19,42	p<0.01**	
			Interaction (F1 X F2)	F=10.22	19,42	p<0.01**	
		PLSD Post-hoc test	CORT *vs* Veh (Target hole, +8 hole)			p<0.01**	
			CORT *vs* Veh (Opposite hole, -1/+1/+9 holes)			p<0.05*	
**Weight**	Weight gain	One-way ANOVA with repeated measures	Factor 1 Treatment	F=7.83	1,19	p<0.01**	S1A
			Factor 2 Time	F=181.79	5,19	p<0.01**	
			Interaction (F1 X F2)	F=19.77	5,19	p<0.01**	
		PLSD Post-hoc test	CORT *vs* Veh, Week 4			p<0.05*	
			CORT *vs* Veh, Week 5 and 6			p<0.01**	
**Coat state**	Score	One-way ANOVA with repeated measures	Factor 1 Treatment	F=108.10	1,19	p<0.01**	S1B
			Factor 2 Time	F=109.36	5,19	p<0.01**	
			Interaction (F1 X F2)	F=16.47	5,19	p<0.01**	
		PLSD Post-hoc test	CORT *vs* Veh (Week 2 to week 6)			p<0.01**	
**Open Field**	Time in center	*t*-test	CORT *vs* Veh	F=5.61	1,38	p<0.05*	S1C
	Ambulatory distance	*t*-test	Factor treatment	F=2.78	1,38	p>0.1	S1D
**Splash test**	Grooming duration	*t*-test	CORT *vs* Veh	F=18.63	1,38	p<0.01**	S1E

Legend: CORT: corticosterone; Veh: Vehicle

Figure S1

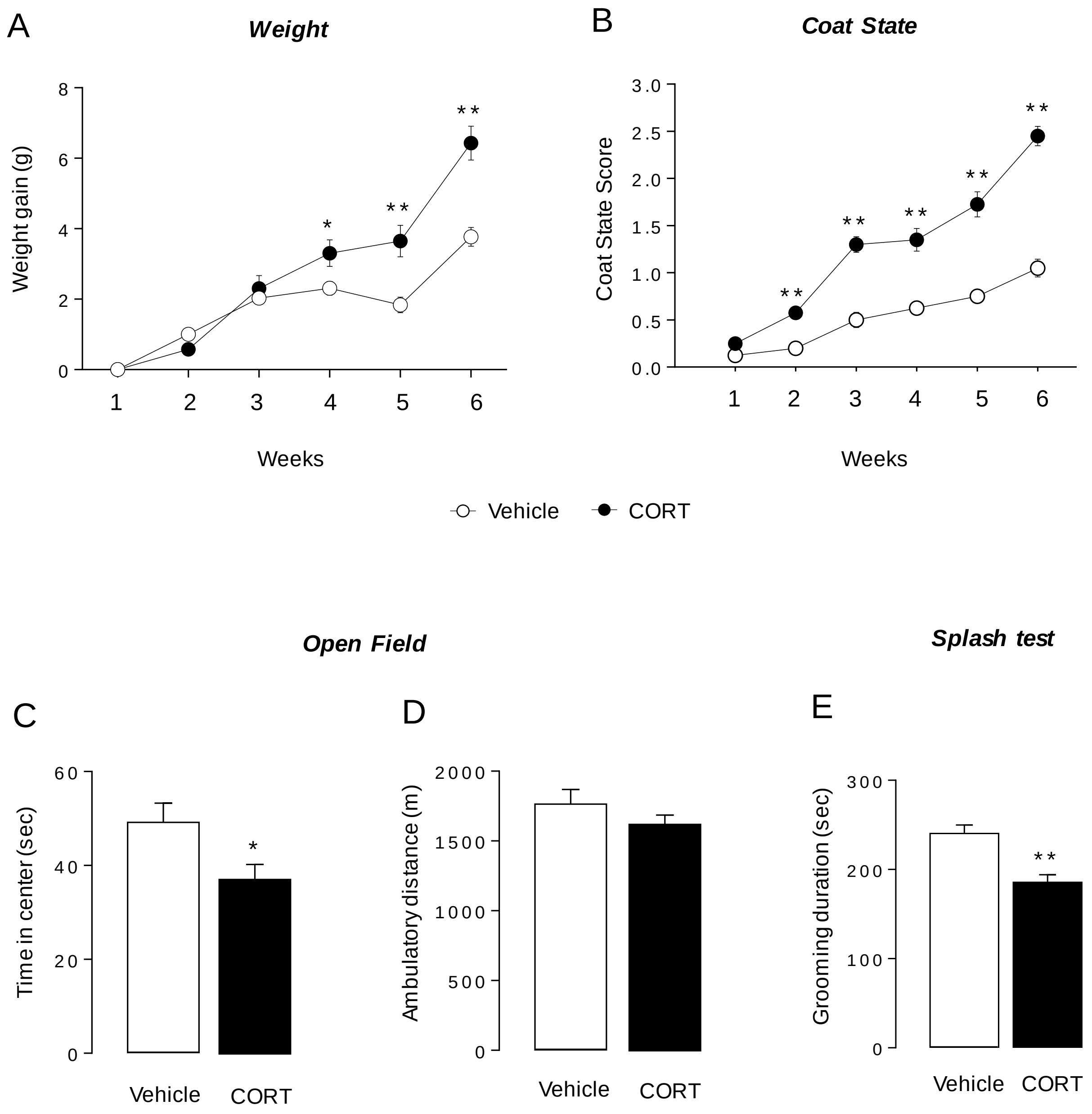
A
Weight
Weight gain (g)
8
6
4
2
0
* *
*
* *
1
2
3
4
5
6
Weeks
B
Coat State
Coat State Score
3.0
2.5
2.0
1.5
1.0
0.5
0.0
* *
* *
* *
* *
* *
1
2
3
4
5
6
Weeks
Vehicle
CORT
Open Field
Splash test
C
Time in center (sec)
60
40
20
0
*
Vehicle
CORT
D
Ambulatory distance (m)
2000
1500
1000
500
0
Vehicle
CORT
E
Grooming duration (sec)
300
200
100
0
* *
Vehicle
CORT

Figure S2

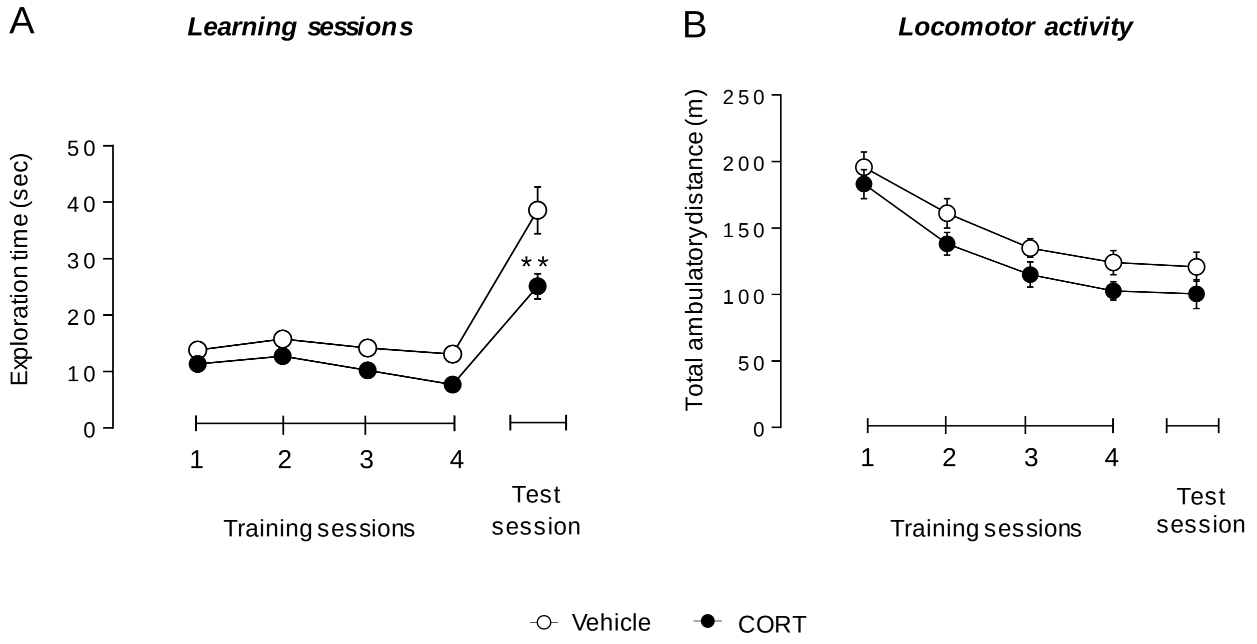
A
Learning sessions
Exploration time (sec)
0
10
20
30
40
50
**
1
2
3
4
Test session
Training sessions
B
Locomotor activity
Total ambulatory distance (m)
0
50
100
150
200
250
1
2
3
4
Test session
Training sessions
Vehicle
CORT

Figure S3

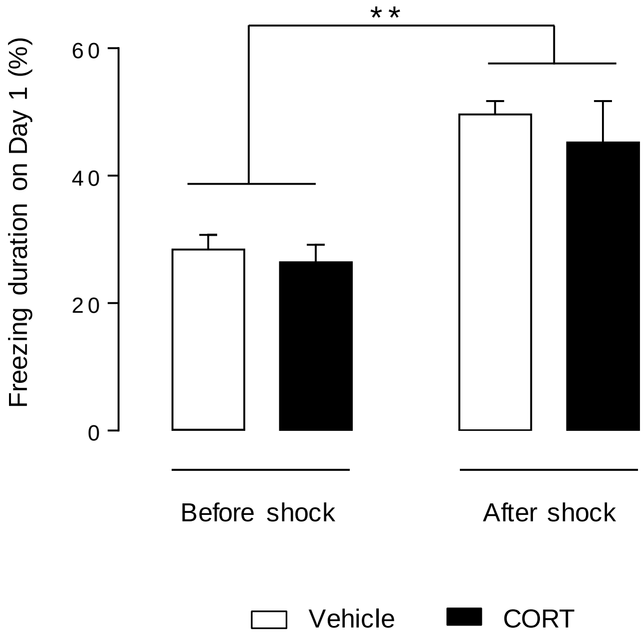
**
60
40
20
0
Freezing duration on Day 1 (%)
Before shock
After shock
Vehicle
CORT

Figure S4

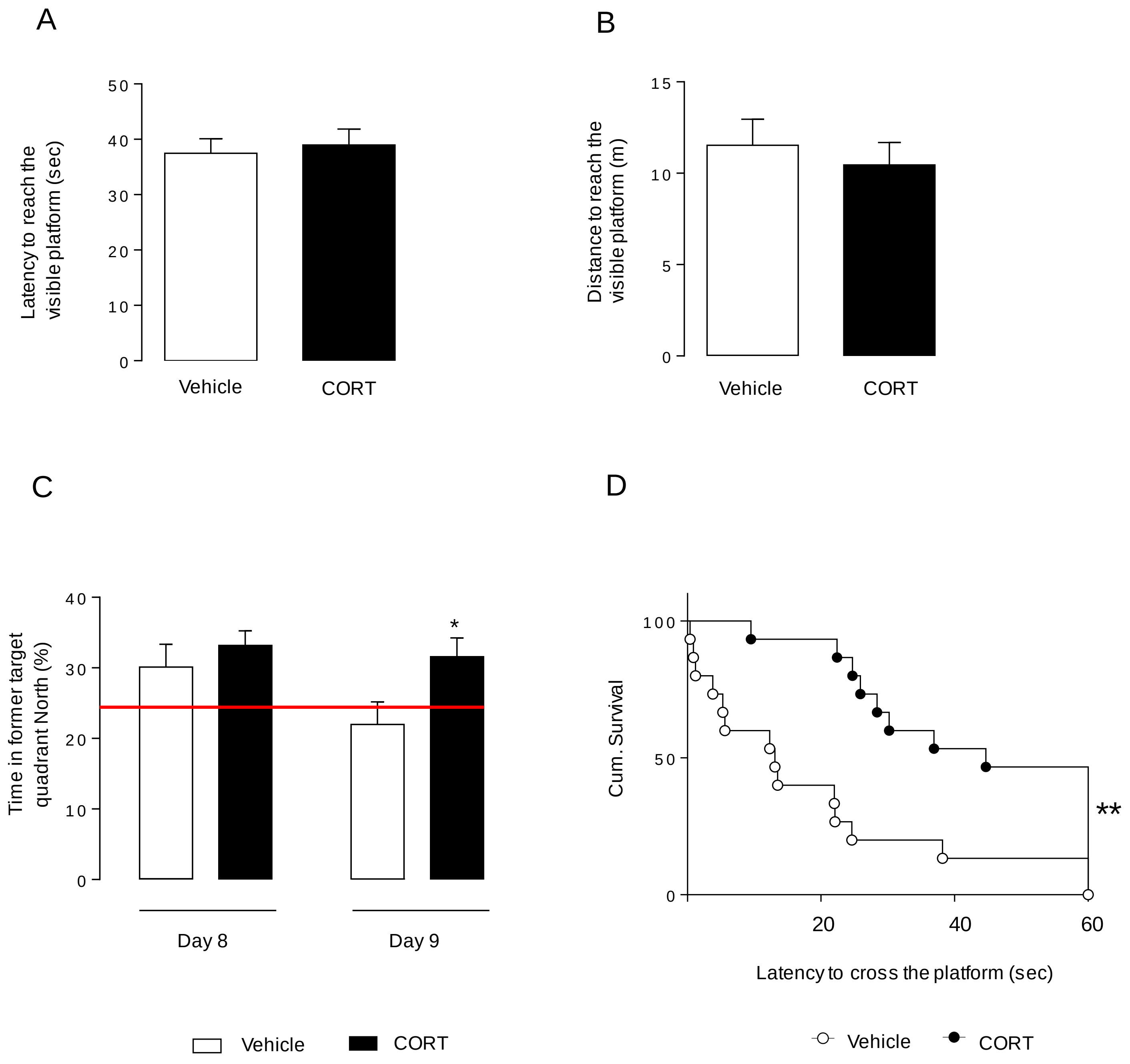
A
Latency to reach the visible platform (sec)
0
10
20
30
40
50
Vehicle
CORT
B
Distance to reach the visible platform (m)
0
5
10
15
Vehicle
CORT
C
Time in former target quadrant North (%)
0
10
20
30
40
*
Day 8
Day 9
Vehicle
CORT
D
Cum. Survival
0
50
100
20
40
60
**
Latency to cross the platform (sec)
Vehicle
CORT

Figure S5

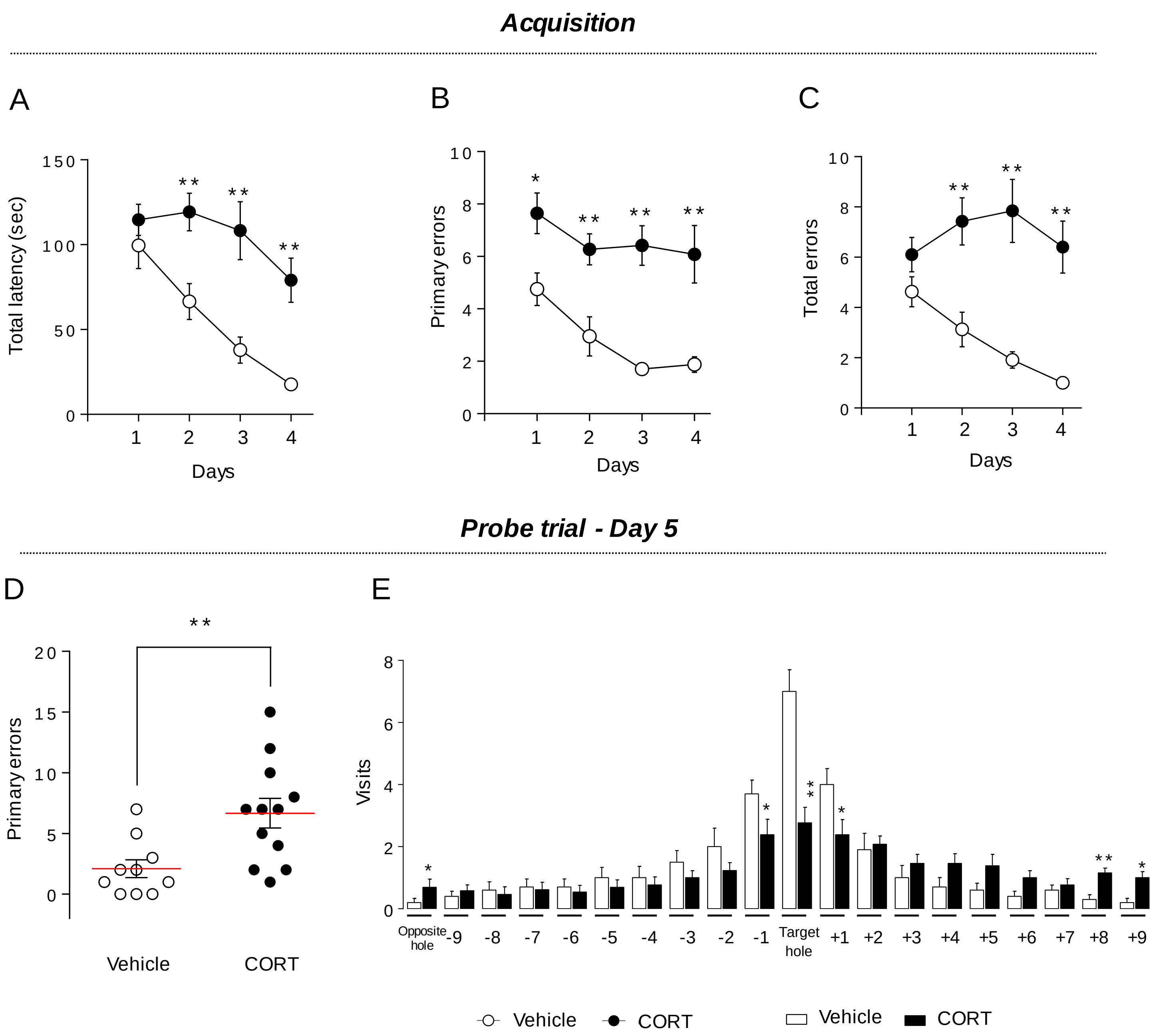

Acquisition
A
B
C
150
100
50
0
Total latency (sec)
1
2
3
4
Days
10
8
6
4
2
0
Primary errors
Total errors
* *
*
Probe trial - Day 5
D
E
20
15
10
5
0
Primary errors
Vehicle
CORT
8
6
4
2
0
Visits
Opposite hole
-9
-8
-7
-6
-5
-4
-3
-2
-1
Target hole
+1
+2
+3
+4
+5
+6
+7
+8
+9
Vehicle
CORT
Vehicle
CORT
